# Expression of the transcription factor PU.1 induces the generation of microglia-like cells in human cortical organoids

**DOI:** 10.1038/s41467-022-28043-y

**Published:** 2022-01-20

**Authors:** Bilal Cakir, Yoshiaki Tanaka, Ferdi Ridvan Kiral, Yangfei Xiang, Onur Dagliyan, Juan Wang, Maria Lee, Allison M. Greaney, Woo Sub Yang, Catherine duBoulay, Mehmet Hamdi Kural, Benjamin Patterson, Mei Zhong, Jonghun Kim, Yalai Bai, Wang Min, Laura E. Niklason, Prabir Patra, In-Hyun Park

**Affiliations:** 1 Department of Genetics, Yale Stem Cell Center, Yale School of Medicine, New Haven, CT 06520 USA; 2 Department of Neurobiology, Harvard Medical School, Boston, MA 02115 USA; 3 Department of Anesthesiology, Yale School of Medicine, New Haven, CT 06520 USA; 4 Department of Molecular, Cellular, Developmental Biology, Yale University, New Haven, CT 06520 USA; 5 Department of Biomedical Engineering, Yale University, New Haven, CT 06520 USA; 6 Department of Biology, Colby College, Waterville, ME 06901 USA; 7 Department of Cell Biology, Yale Stem Cell Center, Yale School of Medicine, New Haven, CT 06520 USA; 8 Department of Pathology, Yale School of Medicine, New Haven, CT 06520 USA; 9 Interdepartmental Program in Vascular Biology and Therapeutics, Department of Pathology, Yale School of Medicine, New Haven, CT 06520 USA; 10 Department of Biomedical Engineering, University of Bridgeport, Bridgeport, CT 06604 USA; 11 Present Address: Department of Medicine, Maisonneuve-Rosemont Hospital Research Centre, University of Montreal, Montreal, Quebec H1T 2M4 Canada

**Keywords:** Reprogramming, Differentiation, Cellular neuroscience, Genetic engineering

## Abstract

Microglia play a role in the emergence and preservation of a healthy brain microenvironment. Dysfunction of microglia has been associated with neurodevelopmental and neurodegenerative disorders. Investigating the function of human microglia in health and disease has been challenging due to the limited models of the human brain available. Here, we develop a method to generate functional microglia in human cortical organoids (hCOs) from human embryonic stem cells (hESCs). We apply this system to study the role of microglia during inflammation induced by amyloid-β (Aβ). The overexpression of the myeloid-specific transcription factor PU.1 generates microglia-like cells in hCOs, producing mhCOs (microglia-containing hCOs), that we engraft in the mouse brain. Single-cell transcriptomics reveals that mhCOs acquire a microglia cell cluster with an intact complement and chemokine system. Functionally, microglia in mhCOs protect parenchyma from cellular and molecular damage caused by Aβ. Furthermore, in mhCOs, we observed reduced expression of Aβ-induced expression of genes associated with apoptosis, ferroptosis, and Alzheimer’s disease (AD) stage III. Finally, we assess the function of AD-associated genes highly expressed in microglia in response to Aβ using pooled CRISPRi coupled with single-cell RNA sequencing in mhCOs. In summary, we provide a protocol to generate mhCOs that can be used in fundamental and translational studies as a model to investigate the role of microglia in neurodevelopmental and neurodegenerative disorders.

## Abstract (web summary)

The study of human microglia function in health and disease is limited by the availability of sound models. Here, the authors develop a method to generate functional microglia in human cortical organoids and investigate the role of human microglia during amyloid beta1-42- induced inflammation.

## Introduction

Microglia are the resident macrophages in the central nervous system (CNS). Their primary functions are to perform immune surveillance in the brain and facilitate brain network formation by regulating neuronal survival, synapse formation, elimination of apoptotic cells, and programmed cell death^1–3^. Recent studies have demonstrated a critical link between impaired microglial function and neurodevelopmental (i.e., Autism or Schizophrenia) and neurodegenerative (i.e., Alzheimer’s disease, AD) disorders^4–6^. Therefore, to investigate the human microglia functions in the context of these diseases, it is essential to develop a model system that reproduces functional microglia in the human brain.

3D brain organoid technology presents an innovative platform to investigate how the human brain develops and experiences neurological diseases^7^. Brain organoids are made either by guided^8,9^ methods using small molecules or unguided^10^ protocols based on the intrinsic neuroectodermal differentiation of pluripotent stem cells (PSCs). However, most brain organoids derived by the directed neuroectoderm differentiation do not contain the mesodermal lineage cells, such as myeloid microglia or endothelial cells^11–13^. Thus, microglia-like cells or progenitors were directly differentiated from hESCs or isolated from primary tissue and co-cultured with brain organoids to study their functions^14–16^. Recently, Ormel et al. reported microglia within the organoids generated through the modified unguided protocol^17^. However, the distribution and the number of functional microglia in these organoids are highly variable due to spontaneous and stochastic features of unguided differentiation. Overall, it is important to produce microglia in brain organoids in a highly reproducible manner to understand the function of these cells in the human brain and dissect their role in the disease state. Here, we develop a method to generate hCOs with the functional microglia from hESCs.

## Results

### Induction of microglia via PU.1 expression under different conditions

Previous studies demonstrated that an ETS-domain transcription factor called *Pu.1* (or *Spi1*) is critical in developing cells into the myeloid lineage, including microglia, and that overexpression of *Pu.1* reprograms the various cell types into myeloid cells^18–20^. Here, we examined whether the PU.1-induced cells in hCOs could differentiate into functional microglia. We utilized the BC4 hESC line expressing rTTA in a doxycycline-inducible manner to induce PU.1 using the lentivirus expressing eGFP. We tested two conditions: embryoid body (EB) differentiation and neuron differentiation conditions (Supplementary Fig. 1a, b). In both settings, the 5-day PU.1-induction dramatically increased the expression of microglia markers such as IBA1 and limited induction of SALL1, as described previously^21^. In contrast, the expression of colony-stimulating factor 1 (*Csf1r*, *Cd115*) regulating the microglia survival, differentiation, and proliferation, and other microglia markers such as *Tmem119*, *P2ry12* were not increased (Supplementary Fig. 1c). This limited induction of microglia markers was most likely caused by the short introduction of PU.1 and in vitro settings related to downregulation as described earlier^22^. Immunostaining (IF) for IBA1 further revealed the presence of microglia-like cells in the differentiating cells that expressed PU.1 for 5 days under either EB or neuron differentiation conditions (Supplementary Fig. 1d). Overall, these data demonstrate that PU.1 expression directs the differentiating hESCs into myeloid cells even in neuroectoderm differentiation conditions.

### Engineering of hCOs with functional microglia (mhCO)

**Fig. 1:**
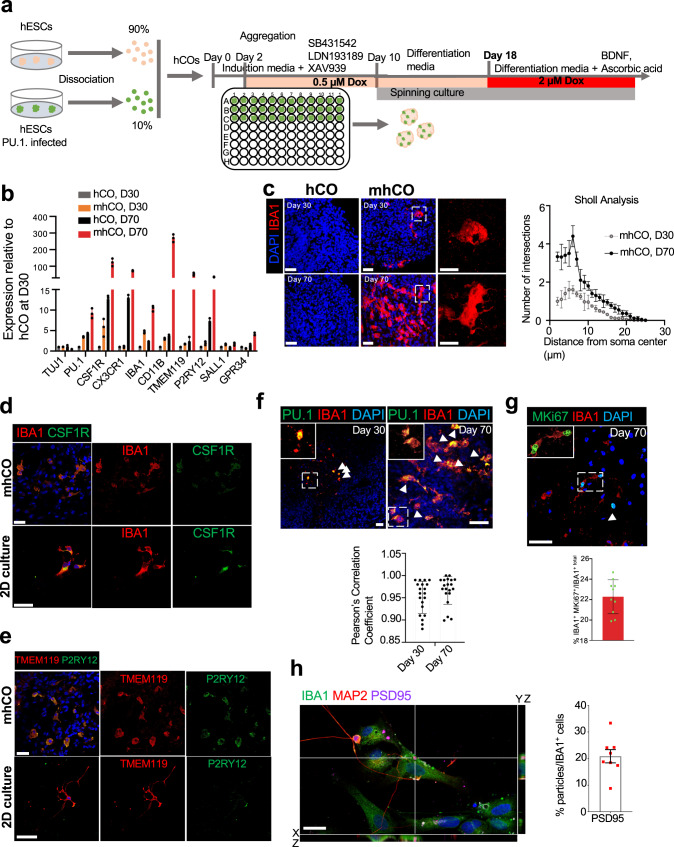
Characterization of microglia-like cells in mhCOs. **a** Schematic for generating mhCOs. 10% of PU.1-infected hESCs were mixed with 90% parental HES3 hESCs, and PU.1 priming and full induction were performed on day 2 and 18, respectively. **b** Expression of microglia-related genes from control hCOs and mhCOs (30-day and 70-day old). Gene expression was measured relative to control organoids on day 30 and normalized to *β-Actin*. Data represent the mean ± SEM (*n* = 5, three independent batches). **c** Left, immunostaining for IBA1 reveals the production of microglia-like cells in sectioned-mhCOs at days 30 and 70. IBA1^+^ cells were not found in control hCOs. Right, Sholl analysis of IBA1 + microglia-like cells from mhCOs at different time points. Data represent the mean ± SEM (*n* = 5 organoids from three independent differentiation replicates of two hESCs lines). **d** and **e** Immunostaining of mhCOs at day 70 and isolated microglia co-cultured with neurons (2D) for IBA1 and CSF1R (**d**) TMEM119 and P2RY12 (**e**). Representative images were shown (*n* = 5, from two independent batches). **f** Top, co-expression of PU.1 and IBA1 in hCOs and mhCOs at day 30 and 70. Bottom, quantification of Pu.1-derived IBA1 microglia-like cells. Data represent the mean ± SEM (*n* = 5 organoids from three independent differentiation replicates of two hESCs lines). Bottom, Pearson’s correlation coefficient of IBA1 with PU.1 in mhCOs at days 30 and 70. **g** Top, co-immunostaining for Ki67 and IBA1 in mhCOs at day 70. Bottom, quantification of proliferating IBA1 microglia-like cells. Data represent the mean ± SEM (*n* = 5 organoids from three independent differentiation replicates of two hESC lines). **h** Left, high-resolution imaging showed microglia isolated from mhCOs at day 90 and co-cultured D90 cortical neurons for 3 days contained inclusions of PSD95. Right, quantification of PSD95 particles in IBA1^+^ microglia-like cells. Data represent the mean ± SEM (*n* = 8, from three independent differentiation replicates of hESCs lines). The scale bar represents 50 μm in **c**–**g** and 20 μm in **h**.

We further tested whether overexpression of PU.1 in the developing cortical organoids could form myeloid cells that function as microglia. Since microglia account for 5–15% of mature brain cells^23–25^, we mixed 10% of PU.1-infected BC4 hESCs with 90% parental hESCs to generate hCOs as described before (Fig. 1a)^26^. In our previous attempt to incorporate endothelial cells (ECs) into hCOs, the partial induction of ETV2 at an early stage, followed by a full induction at day 18, was optimal in converting differentiating cells into ECs^27^. We applied a similar induction scheme in over-expressing PU.1 in hCOs (Fig. 1a). 3D live imaging of hCOs revealed the organization of the GFP^+^ cells with an amoeboid cell shape at 30-day-old hCOs (Supplementary Fig. 1e). 70-day-old hCOs contain a dramatically higher number of cells displaying the amoeboid cell shape than 30-day-old hCOs (Supplementary Fig. 1e). Consistent with the confocal imaging, PU.1-induced hCOs exhibit a higher expression of microglial markers at day 70 (Fig. 1b). Additionally, PU.1-induced hCOs showed increased expression of the microglial markers *Iba1*, *Csf1r*, *Cd11b*, *Cx3cr1*, *Tmem119*, and *P2ry12* (Fig. 1b). These results suggest that hCOs with the forced PU.1 expression contain microglia-like cells. We named these organoids microglia-like cells-containing hCOs (mhCOs).

To investigate whether the microglia-like cells produced by PU.1-induction at mhCOs function like in vivo microglia, we assessed microglial characteristics in 30-, 70-, or 90-day -old mhCOs. The immunostaining analysis for a microglia marker, IBA1, indicated the generation of microglia-like cells in mhCOs but not in control hCOs (Fig. 1c). On day 30, mhCOs contained many microglia-like cells with amoeboid morphology, whereas control hCOs lacked these cells. On day 70, more IBA1^+^ microglia-like cells were found in mhCOs, and they displayed an enhanced complexity in ramified morphology with the increased number of branch points characterized by Sholl analysis (Fig. 1c). qPCR results further supported the immunostaining data and showed an elevated expression of *Iba1* in mhCOs compared to control hCOs at either day 30 or day 70 (Fig. 1b, c). As described previously^28^, we sorted GFP^+^, microglia-like cells and cultured them with cortical neurons isolated from the same mhCOs at day 70 to characterize these immune cells in 2D-culture and 3D-culture settings. Co-immunostainings for IBA1 and CSF1R or TMEM119 and P2RY12 indicate that microglia-like cells acquire ramified structures within mhCOs as well as 2D-culture conditions (Fig. 1d, e, Supplementary Movie 1 for hCO, and Supplementary Movie 2 for mhCO). Even though GFP^+^ microglia-like cells became more ramified in 2D settings than 3D systems, mhCOs possess a higher expression of microglia-like cells P2RY12, suggesting the 3D-environment resembling the physiological conditions (Fig. 1b, d, e)^22^. Notably, IBA1^+^ microglia in mhCOs are originated from PU.1-expressing GFP^+^ cells (Fig. 1f and Supplementary Fig. 1f). On day 70, 22% of microglia-like cells express both IBA1 and MKI67, revealing the presence of proliferating microglia (Fig. 1g). We then investigated the function of microglia on synapse pruning. Indeed, co-immunostaining uncovered the presence of PSD95 puncta within IBA1^+^ microglia, indicating the presence of functional microglia engulfing or contacting the neuronal synaptic structures from mhCOs (Fig. 1h). Based on the detection of neuronal activity markers *Fos* and *Npas4* with single-molecule RNA fluorescent in situ hybridization, we did not observe any difference in neuronal activity of mhCO and hCO at day 70 (Supplementary Fig. 2a). We further confirmed that both hCOs and mhCOs at days 70–80 and 110–120 contained functional neurons with calcium surges utilizing the genetically encoded GCAMP6s (Supplementary Fig. 2b, c). Moreover, no changes in either spontaneous firing amplitude or frequency were observed between hCOs and mhCOs at days 70–80, indicating that the introduction of microglia-like cells does not influence the functional properties of neurons in organoids earlier time point (Supplementary Fig. 2b). On the other hand, neurons from mhCOs displayed more and patterned neuronal activity with enhanced spontaneous firing frequency at days 110–120 (Supplementary Fig. 2c). This data suggests the regulatory role of microglia on the maturation of the neural networks in organoids at later development. This finding is consistent with previous organoid models co-cultured with either human microglia isolated from cortex^15^ or induced pluripotent stem cell-derived erythromyeloid progenitors^16^. We further noted the presence of microglia-like cells on the surface of mhCOs but not on the surface of hCOs by using scanning electron microscopy (Supplementary Fig. 2d). Regarding corticogenesis, no significant difference in the formation of cortical layers was noted in mhCOs compared to hCOs, as demonstrated by the presence of the intermediate progenitors, deeper and upper cortical layer neurons characterized by the expression of TBR2, CTIP2, or SATB2 (Supplementary Fig. 2e). These data suggest that the generation of microglia-like cells by PU.1-induction has little impact on cortical development. Collectively, PU.1 induction directs cells into microglia-like cells in hCOs.

### PU.1-induced microglia-like cells from hESCs engrafting mouse brain

We next examined the in vivo function of PU.1-induced microglia-like cells in the mouse brain. Microglia-like cells from hESCs by PU.1 induction were transplanted into the brain of an immune-deficient mouse and treated with Aβ-oligo (Supplementary Fig. 2f). After 3 weeks of transplantation, immunostaining for human-specific CD68 and Aβ revealed that PU.1-derived microglia-like cells displayed a complex ramified morphology and were functionally active, responding to oligomeric Aβ (Supplementary Fig. 2g). Collectively, these data suggest that microglia-like cells from PU.1 induction function similarly within the mouse brain environment and human brain organoids.

### A single-cell map of developing mhCOs reveals the presence of microglia-like cells resembling the primary counterparts

**Fig. 2:**
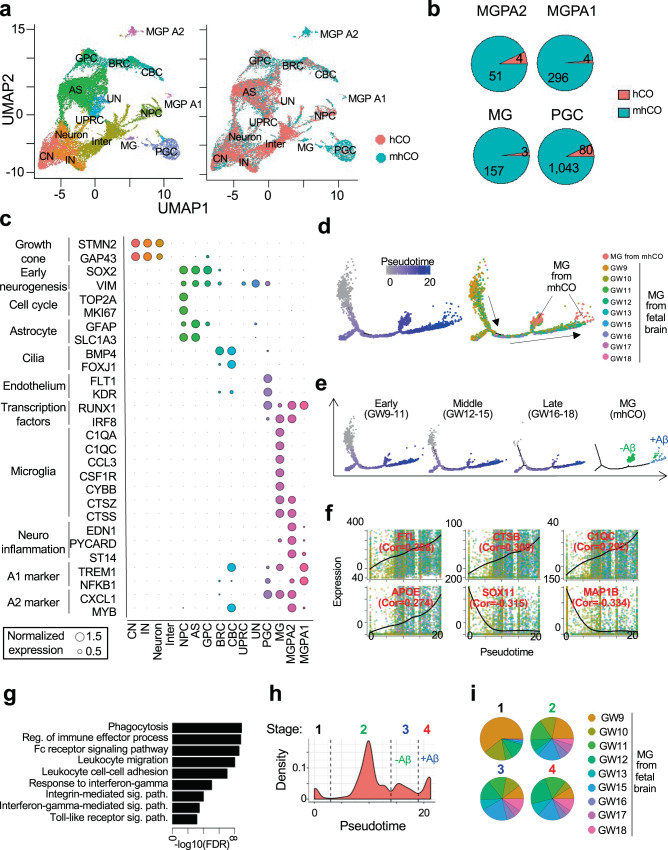
Single-cell map of mhCOs. **a** Uniform Manifold Approximation and Projection (UMAP) plot of single cells from hCOs and mhCOs colored by cell type assignment (left) and organoid type (right). CN cortical neuron, IN interneuron, Neuron: non-committed neuron, Inter intermediate, NPC neuronal progenitor cell, AS astrocyte, GPC glia progenitor cells, BRC BMP responsible cell, CBC cilia-bearing cell, UPRC unfolded protein response-related cell, UN unassigned cell, PG proteoglycan-expressing cell, MG microglia, MGPA1 microglia progenitor-2 possessing keratin-enriched gene signatures, MGPA2 microglia progenitor-1 exhibiting claudin-enriched gene signatures. Data depicts results from 26,962 cells. **b** Pie chart representing the cell count from hCO and mhCO in MG, MGPA1, MGPA2, and PGC clusters. **c** Circle plot showing relative expression of cell type-specific markers across cell types. Circle size represents normalized gene expression. **d–i** Integrative analyses of scRNA-seq of microglia from mhCOs and human fetal brains. **d** Trajectory plots depicting pseudotime (left) and age (right). **e** Trajectory plots separated by the human fetal brain at each developmental stage and organoid. The treatment of Aβ further separated organoid-derived microglia cells. **f** Gene expression profiles over pseudotimes for representative differentially expressed genes. **g** Significant GO terms of genes elevated with pseudotimes. **h** Histogram of pseudotime. Cells were categorized into four distinct stages with thresholds that are shown by dashed lines. **i** Ratio of fetal brain ages across the developmental stages.

We performed single-cell RNA-sequencing (scRNA-seq) in the 10X Genomics platform to examine the lineage specification employed by PU.1 induction during cortical organoid development. After quality control, a total of 13,416 and 13,546 cells were analyzed from hCOs and mhCOs, respectively (Fig. 2a). Cell clusters were then systematically assigned to 14-cell types, including cortical neurons, interneurons, astrocytes, and other previously defined cell types (Supplementary Fig. 3 and see the “Methods” section)^29^. Interestingly, mhCOs uniquely produced four cell types, including microglia cluster (MG) with high expression of the complement/chemokine system (e.g., *C1qa*, *C1qc*, *Ccl3/4*), microglia precursor cluster A1 (MGPA1) expressing yolk sac progenitor markers (*Trem1* and *Nfkb1*) and microglia precursor cluster A2 (MGPA2) expressing myeloid progenitor markers (*Cxcl1*, *Myb*, and *Irf8*) and PGC cluster highly expressing proteoglycans (Fig. 2a, b). Comparative analysis with the reference transcriptome and unique cell markers validated that the cell cluster with the immune system genes is microglia (Supplementary Fig. 3c, e)^30^. PGCs were previously shown to transdifferentiate into endothelial cells by a defined transcription factor, ETV2^27^. In mhCO-derived cells, we also detected the enhanced expression of endothelial (*Flt1* and *Kdr*) and pericyte markers (*Acta2* and *Pdgfrb*) in PGC (Fig. 2c). This data suggests that PU.1 promotes mesoderm-derived cell types. Indeed, MGPA1 and A2 clusters also expressed some of the microglia-related genes (e.g., *Ctss*, *Lcp1*) (Fig. 2c). Notably, both cell types are also detected in single-cell transcriptome from in vivo human fetal brain samples (Supplementary Fig. 3f)^31^. Their gene signatures are significantly enriched in in vitro-derived microglia precursors, suggesting that these cell types are potential precursors of microglia cells (Supplementary Fig. 4a)^32^. Myeloid progenitors in the yolk sac migrated to CNS and developed into immature A1 and A2 cells, becoming microglia^18^. We further compared the transcriptome of MGPA1, MGPA2, and MG clusters with yolk sac A1, A2 precursors, and human fetal microglia. Indeed, MGPA1 and MGPA2 clusters acquire expression of A1 (*Trem1* and *Nfkb1*) and A2 (*Cxcl1*, *Myb*, and *Irf8*) markers, respectively (Fig. 2c). Thus, these results support our hypothesis that MGPA1 and MGPA2 are potential precursors during microglia development.

Comparative analysis of the global gene expression profile revealed that mhCOs displayed a significant induction of genes involved in immune cell differentiation, toll-like receptor signaling, and NF-κB signaling (Supplementary Fig. 4b–d). Some of these genes are also related to inflammatory signaling pathways indicating that microglia within mhCOs respond to the internal stress, most likely apoptotic cells due to the limited diffusion of nutrients and oxygens at day 90 organoids^33,34^. These results indicate that PU.1 mediates the lineage commitment into microglia-like cells during cortical organoid development.

In one of the previous studies by Ormel et al., authors detected the microglia-like cells, defined as oMG (organoid MG), in brain organoids made by an unguided protocol^17^. The enrichment of gene signatures of oMG and primary microglia (pMG) was applied to our scRNA-seq data to examine differences between the innately developed oMG and the PU.1-induced microglia (MG). We found that oMG gene signatures were enriched in PGC clusters but depleted in MG clusters (Supplementary Fig. 4e, f). In contrast, pMG gene signatures were enriched in MG clusters and significantly increased with Aβ treatment (Supplementary Fig. 4f, *p* = 4.70−e2 by two-sided *t*-test). These results suggest that PU.1-induction produced microglia-like cells demonstrating a more similar transcriptome profile to primary microglia than oMG.

In the embryonic and early postnatal brain, microglia are heterogeneous and progressively mature^35^. Integrative analysis of scRNA-seq revealed that transcriptional profiles of microglia-like cells from mhCOs are similar to those in the middle and late stages of fetal microglia development (Fig. 2d). Assigned pseudotimes are increased with the gestational week (GW) (Fig. 2e) and orchestrated with activation of immune sensing (Fig. 2f, g). The pseudotime trajectory analysis classified microglia into four distinct developmental stages (Fig. 2h). The PU.1 induction in the brain organoid produced the third stage of microglia-like cells mainly composed of GW12–18 (Fig. 2h, i). Collectively, our results indicate that microglia-like cells in mhCOs recapitulate the transcriptional surveillance of the human fetal brain to pathological hallmarks.

### Lineage commitment of PU.1-eGFP expressing cells and their functions in mhCOs

**Fig. 3:**
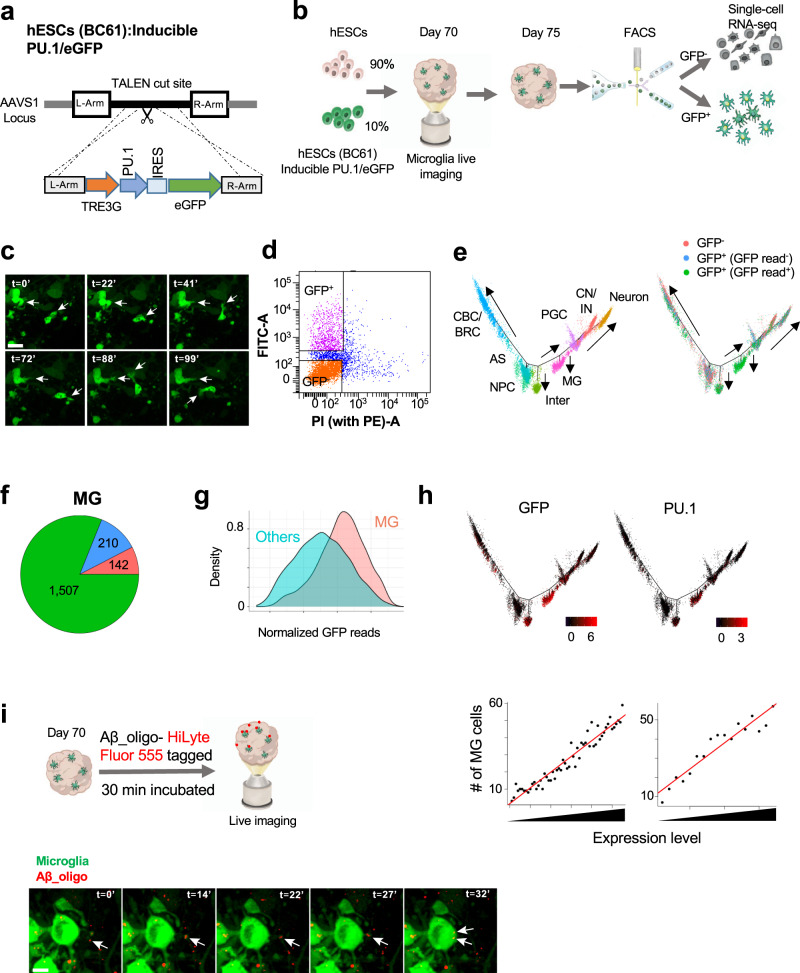
Microglia lineage commitment of GFP-expressing cells and their roles in the organoid (mhCO). **a** A construct to target the AAVS1 locus to generate rtTA-PU.1-IRES-eGFP^+^ hESC line (BC61). **b** Schematic showing the protocol for generating mhCOs by using hESCs (BC61). **c** Time-lapse imaging indicating GFP^+^ microglia over 100 min within mhCOs. Representative images with white arrowheads showing motile microglia. Time is shown in minutes. **d** FACS analysis of dissociated mhCOs at day 75 shows that GFP^+^ and GFP^−^ cells are sorted for performing scRNA-seq. **e** Cell trajectory analysis of the single-cell transcriptome of GFP^+^ and GFP^−^ cells. Cells are colored by trajectory branches and library sources. **f** Ratio of GFP^+^ and GFP^−^ cells in MG clusters. **g** Comparison of GFP expression between MG and other branches (two-sided *T*-test *p* < 2.2e-16). **h** Relationship between GFP/PU.1 expression and MG lineage commitment. (Top) GFP and PU.1 expression are plotted in the cell trajectory map. (Bottom) Correlation between GFP/PU.1 expression and efficiency of MG lineage commitment. Cells are sorted by GFP/PU.1 expression and divided into 100-cells bins. The number of cells in the MG branch is shown in each bin. **i** Top, schematic showing Aβ42-oligo-(HiLyte)-555 treatment on mhCOs. Bottom, a representative image is showing GFP^+^ microglia phagocytosing Aβ (red) in around 32 min within mhCOs. The scale bar represents 20 μm in (**c**) and 10 μm in (**i**). Imaging was repeated in organoids from three independent differentiation experiments with similar results in **c** and **i**.

To address the characterization of heterogeneity of the cells produced via PU.1 induction within mhCOs, we introduced dox-inducible *PU.1-iRES-eGFP* and rtTA cassette into the AAVS1 safe locus to generate hESC line (BC61) with robust transgene expression tagged with GFP (Fig. 3a). As described above, we generated cortical organoids with microglia-like cells (mhCO), mixing 10% of BC61 hESC cells with 90% parental hESC cells and characterized GFP^+^ microglia-like cells via live-imaging (Fig. 3b). Time-lapse imaging experiments up to 2-h showed that GFP+ microglia-like cells acquire diverse morphology, amoeboid and ramified, and remarkably motile and dynamic (Fig. 3c and Supplementary Movie 3). We then extracted GFP^+^ and GFP^−^ cells by fluorescence-activated cell sorting on day 75 to examine how GFP-expressing cells committed into myeloid lineage via performing scRNA-seq (Fig. 3b, d, and Supplementary Fig. 5a). The developmental trajectory segregated cells into eight branches corresponding to microglia, neurons, astrocytes, and other cell lineages (Fig. 3e and Supplementary Fig. 5b). We note that more than half of cells in GFP^+^ populations contain GFP-derived RNA-seq reads, whereas the GFP-derived reads were rarely detected in GFP^−^ cells (Supplementary Fig. 5c). We suppose that GFP^+^ cells without GFP-derived reads may induce the transgene with a lower level than those with GFP-derived reads. Notably, most cells in the MG branch were composed of cells from the GFP^+^ population with GFP-derived reads (Fig. 3e, f). GFP expression in the MG branch is significantly higher than that in other branches (Fig. 3g). Though some GFP^+^ cells with GFP-derived reads were classified into different cell types (e.g., intermediate), GFP^−^ cells and GFP^+^ cells without GFP-derived reads were barely differentiated into MG lineage. Since exogenous PU.1 does not contain poly-A tail, PU.1 reads are mainly derived from endogenous PU.1, usually silenced in non-MG cell types in the brain. We found that the vast majority of PU.1-expressing cells contain GFP-derived reads (Supplementary Fig. 5d), and endogenous PU.1 expression level was positively correlated with GFP expression (Spearman correlation = 0.520). Interestingly, cells with higher GFP and endogenous PU.1 levels are more likely to be differentiated into microglia (Fig. 3h). Overall, our results suggest that the substantial expression of PU.1 assists microglia fate specification in the organoid.

We next examined whether GFP^+^ microglia-like cells display phagocytotic functions by performing 4D (*x*, *y*, *z*, and *t*) imaging of live mhCO. To visualize the response of microglia towards Aβ_1-42_-oligo inflammation, we treated mhCOs with 1 μM of Aβ_1-42_-oligo-(HiLyte)-555 for 30 min (Fig. 3i). We then performed live imaging of GFP^+^ microglia-like cells and Aβ_1-42_-oligo-(HiLyte)-555 and observed several GFP^+^ microglia-like cells already captured Aβ_1-42_-oligo-(HiLyte)-555 found in the soma of microglia. As a particular event, microglia-like cells migrated toward Aβ, extended processes, and captured Aβ for phagocytosis lasting in around 30 min (Fig. 3i and Supplementary movie 4). Thus, GFP^+^ microglia-like cells rapidly process Aβ in mhCOs resembling quick (in minutes) microglial actions towards Aβ in mice brains^36^. Taken together, PU.1 drives the generation of microglia-like cells with high motility and functionality in mhCOs.

### Microglia within mhCOs recapitulates the actions of primary microglia towards amyloid-β

**Fig. 4:**
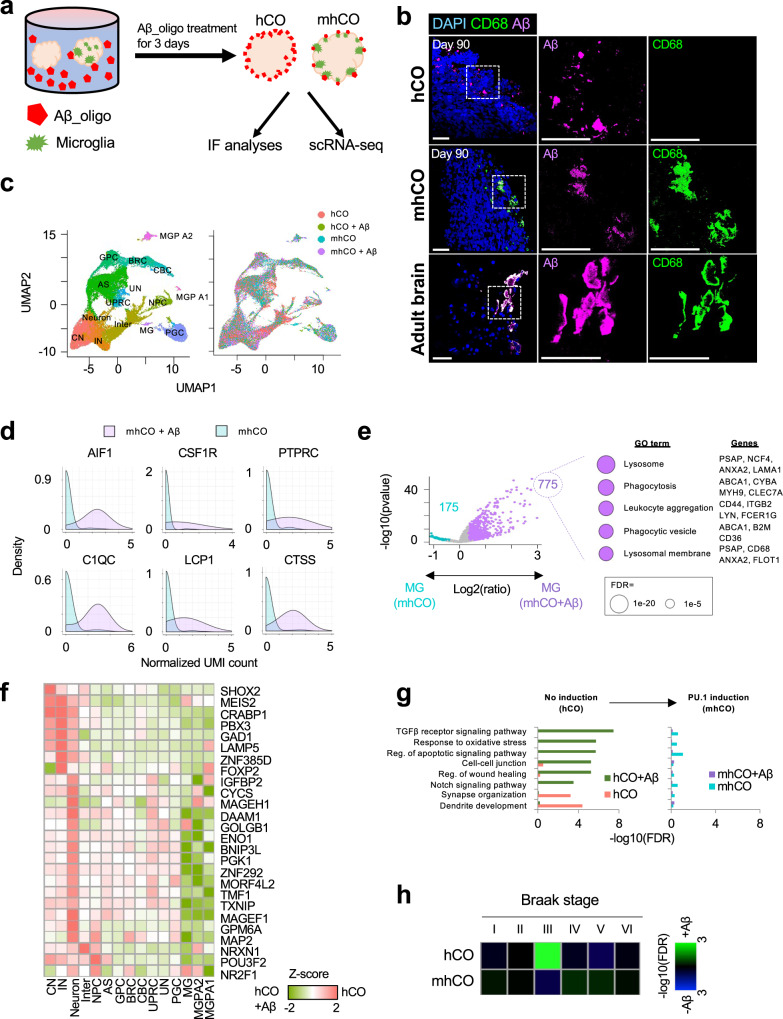
Effects of Aβ treatment on cortical organoids. **a** Experimental design to test the effect of Aβ_oligo treatment on control hCOs and mhCOs. **b** Co-Immunostaining for CD68 and Aβ in Aβ-treated control hCOs, mhCOs, and the adult human brain. Data are representative images of five organoids from three independent experiments. The scale bar represents 50 μm. **c** UMAP plot of single cells from non- and Aβ-treated hCOs and mhCOs colored by cell type assignment (left) and organoid type (right). Data depicts results from 49,867 cells. **d** Histogram representing significant gene induction related to microglia activation in non- and Aβ-treated mhCOs. *p* = 2.51e−11 (AIF1), 3.64e−4 (CSF1R), 2.79e−7 (PTPRC), 2.04e−10 (C1QC), 9.52e−7 (LCP1), and 1.42e−11 (CTSS) with two-sided *t*-test. **e** Volcano plot representing differential gene expression in microglia between non- and Aβ-treated mhCOs (left). 775 up- and 175 down-regulated genes are shown by purple and turquoise color, respectively. The other 31,788 genes are shown by gray color. The top five significant GO terms of upregulated genes in Aβ-treated microglia were shown by circle size (right). A two-sided *t*-test was used for comparison. **f** Heatmap representing the down-regulation of neuronal developmental genes in hCOs with Aβ treatment. Relative expression value in non-treated hCO to Aβ-treated hCOs was normalized as *Z*-score. **g** GO enrichment of global differentially expressed genes between non- and Aβ-treated organoids. Left and right bar plots were obtained from hCO and mhCO, respectively. **h** GSEA of Braak stage-specific gene signatures in Aβ-treated hCOs and mhCOs. The enrichment and depletion were visualized by blue and green colors, subsequently.

Microglia play critical roles in inflammation within the CNS, such as a rapid response to inflammatory signaling^4^. We investigated the function of microglia under Aβ_1-42_-oligo treatment (for 72 h), a pro-inflammatory trigger (Fig. 4a). The Aβ_1-42_-oligo treatment led to a dramatic change in the surface of control hCOs. Surprisingly, Aβ_1-42_-oligo had little effect on the surface of mhCOs (Supplementary Fig. 6a). Apoptosis in hCOs induced by Aβ_1-42_-oligo was prominent as represented by deterioration on the exterior of hCOs, while mhCOs exhibited reduced apoptotic areas on their surfaces (Supplementary Fig. 6a). Notably, mhCOs treated with Aβ_1-42_-oligo displayed even fewer apoptotic areas than non-treated mhCOs, suggesting that Aβ_1-42_-oligo further enhanced the scavenging function of the microglia-like cells in mhCOs (Supplementary Fig. 6b). Surprisingly decreased apoptotic regions in mhCOs under Aβ-treatment indicate microglia cells’ regular or extensive activation. Although the number of microglia is limited in mhCOs, the incubation of Aβ_1-42_-oligo for 3 days may keep them fully activated and direct them to eliminate apoptotic cells. Co-staining of Aβ with CD68 demonstrated a co-localization of microglial phagocytotic markers with Aβ oligos (Fig. 4b, Supplementary Movie 5 for hCO, Supplementary Movie 6 for mhCO). Indeed, staining for IBA1, LAMP1 and Aβ revealed that dystrophic neurites marked by LAMP1 surrounded Aβ oligos (Supplementary Fig. 6c). Notably, microglia frequently engage with Aβ oligos which may show the protective role of microglia on Aβ-induced neurotoxicity (Supplementary Fig. 6c). Similar to actions of microglia towards Aβ in mhCOs, primary microglia in human tissue engulf the Aβ, characterized by the presence of CD68 (Fig. 4b). The microglia in mhCO and primary human tissue showed that microglia-like cells have processes different from typical microglia surrounding Aβ plaques^36^. The difference may be due to the in vitro response being different from the in vivo condition^37^. These results suggest that microglia-like cells within mhCOs become activated, engulf the Aβ, and scavenge the dead cells.

### A single-cell map of amyloid-β treated organoids uncovers the protective role of microglia

To examine the effects of Aβ on cell differentiation and transcriptional program in hCOs, we further investigated the single-cell transcriptome in cortical organoids treated with Aβ (Fig. 4c). Aβ-treated mhCOs exhibited a significant up-regulation of genes associated with microglial activation (*Aif1*, *C1qc*, *Csf1r*, *Lcp1*, *Ptprc*, and *Ctss*) in MG clusters compared to those in non-treated mhCOs (Fig. 4d). Furthermore, as observed in an AD mouse model^38^, microglia-like cells in Aβ-treated mhCOs showed a high expression of genes involved in the lysosome, phagocytosis, and leukocyte aggregation (Fig. 4e). Thus Aβ treatment led to the induced phagocytic activity of microglia in the organoids. The induction of phagocytic genes was also observed in the AD patient brain compared to the brain from healthy donors (Supplementary Fig. 7a)^39^.

In addition to mhCOs, we also evaluated the effects of Aβ-treatment on hCOs (Fig. 4c). Global transcriptome comparison revealed that the genes related to synapse and dendrite development were significantly downregulated (Fig. 4f). qPCR and immunostaining analyses further confirmed the dysregulation of synaptic and dendritic genes in Aβ-treated hCOs but not in Aβ-treated mhCOs (Supplementary Fig. 7b, c). hCOs showed the abnormal induction of genes for apoptotic, TGFβ, and Notch signaling (Fig. 4g) involved in AD pathogenesis^40,41^. Significantly, the transcriptional dysregulation of dendritic and synapse development and apoptotic genes was significantly attenuated in mhCOs (Fig. 4g). Moreover, genes promoting neuronal differentiation and maturation (*Brn2* (*Pou3f2*), *Crabp1*, and *Foxp2*) as well as glia cell commitment (*Basp1* and *Plp1*) were drastically reduced in hCOs but were protected in mhCOs (Supplementary Fig. 7d). These results suggest that functional microglia in mhCOs rescues the Aβ-mediated cellular and molecular abnormalities in hCOs.

Ferroptosis is a type of cell death caused by iron-dependent lipid peroxidation. Increased iron level is a common feature of neurodegeneration and is related to the progression of AD^42^. Notably, Aβ treatment resulted in the over-expression of ferroptosis mediators, such as *Phkg2*^43^, *Txnrd1*^44^, and *Pparg*^42^, in hCOs but not in mhCOs (Supplementary Fig. 7e). GSEA further confirmed that ferroptosis-associated genes were significantly increased with Aβ treatment in hCOs (Supplementary Fig. 7f). Thus, the presence of functional microglia attenuates Aβ-mediated induction of ferroptosis. We further compared the transcriptome profiles of our Aβ-treated cortical organoids with those from AD patient cortexes collected at different Braak tangle stages^45^. Comparison analysis revealed that Aβ-treated hCOs displayed a significant enrichment of gene signatures of stage III, during which the neurofibrillary lesions invade the neocortex region and patients start to exhibit the clinical symptoms (Fig. 4h)^46^. On the contrary, mhCOs did not show any stage-specific gene signatures (Fig. 4h). Interestingly, Aβ treatment induced the microglia into the fourth stage of fetal microglia that displays transcriptional features of phagocytic microglia (Fig. 2h). Overall, our Aβ-treated brain organoids recapitulate the onset of AD-associated transcriptomic profile, which can be rescued by PU.1-induced microglia-like cells.

### mhCOs provide a valuable tool to examine the role of AD-associated microglia genes

**Fig. 5:**
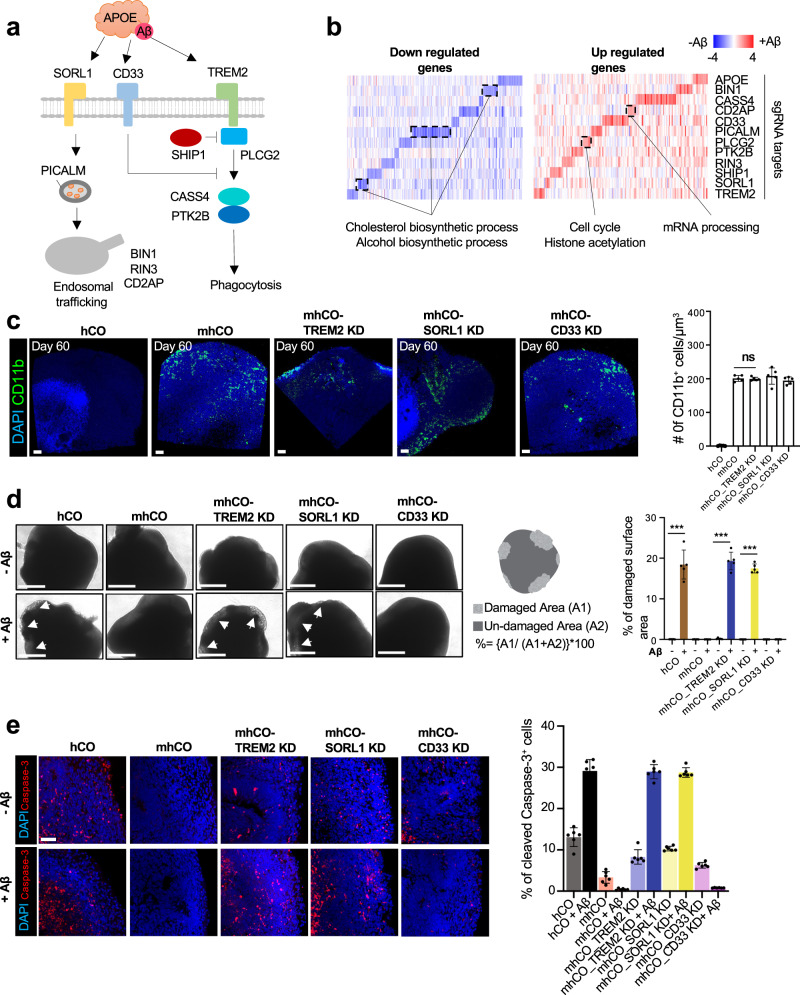
CRISPRi-mediated suppression of AD-genes in MG from mhCOs. **a** Relationship between target genes and pathways for endocytosis, phagocytosis, and degradation. **b** Differential gene expression between non- and Aβ-treated mhCOs in each knockdown. **c** Left, whole-mount immunostaining for CD11b in hCO, mhCO, and mhCOs with CRISPRi-mediated suppression of TREM2, SORL1, or CD33 at day 60. Right, quantification of the number of CD11b+ microglia-like cells/μm^3^. Data represent the mean ± SEM (*n* = 5, from three independent batches, two-sample *t*-test with Welch’s correction). The scale bar represents 100 μm. **d** Left, representative images of control hCO, mhCO, and mhCOs with CRISPRi-mediated suppression of *Trem2*, *Sorl1*, or *Cd33* at 60-day-old with and without Aβ treatment. White arrowheads are showing damaged surfaces. Middle, schematic showing quantification of surface damages induced by Aβ treatment. Right, quantification of % of surface damage on organoids with and without Aβ treatment. Data are representative images of five organoids from three independent experiments. Bars indicate mean and SEM. The unpaired two-tailed *t*-test was used for all comparisons, ***p = 0.000149 for hCO and mhCO_SORL1 KD, ***p = 0.000003 for mhCO_TREM2 KD. The scale bar represents 0.5 mm. **e** Left, cleaved caspase-3 staining of organoids after 60-day culture with and without Aβ treatment. Right, quantification of cleaved caspase-3+/DAPI+ cells in hCOs and mhCOs at days 60 with and without Aβ treatment. Data represent the mean ± SEM (*n* = 6 organoids from three independent differentiation replicates of two hESC lines). The scale bar represents 50 μm.

Previous genome-wide association studies (GWAS) and exome-sequencing have identified several microglia-associated and immune response genes for AD^47,48^. Using PU.1-induced microglia-like cells as a model system, we explored the role of AD-linked genes in responding to Aβ treatment (Fig. 5a). We used CRISPRi (CRISPR interference) coupled with CRISPR droplet sequencing (CROP-seq) format to suppress the expression of these genes and to implement high-throughput genetic perturbation at the single-cell level^49^. We designed a gRNA library to target 12 AD risk genes involved in endocytic trafficking, degradation, and phagocytosis of Aβ_1-42_ (Fig. 5a and Supplementary Table 1). After hESCs stably expressing dead Cas9 (dCas9) were transduced with gRNA library lentivirus and organoids generated, we analyzed the function of AD-risk genes under Aβ_1-42_-oligo treatment via CROP-seq. By comparing transcriptome patterns with and without Aβ_1-42_ treatment, we identified a significant reduction of cholesterol metabolic genes by Aβ_1-42_ treatment in cells that are inhibited for endocytosis-related genes; *Picalm*, *Sorl1*, and *Bin1* (Fig. 5b and Supplementary Fig. 8a). Previous GWAS studies also stressed that genes associated with endocytosis were linked with the development of late-onset AD and may lead to the perturbation of cholesterol homeostasis^50,51^. In addition, a low quantity of cholesterol promotes the formation of voltage-independent ion channel pores with Aβ_1-42_ and results in disruption of Ca^2+^ homeostasis and membrane potential in neurons^52^. This result suggests that pooled CRISPRi screening from mhCOs provides a valuable tool to better understand the role of microglia in AD.

In addition to pooled CRISPRi screening, we further explored the role of three AD-linked microglia genes (*Trem2*, *Cd33*, and *Sorl1*) by generating mhCOs with targeted suppression of each gene (Supplementary Fig. 8b–d). Two gRNAs for each target were tested for the efficacy of CRISPRi in hESCs (Supplementary Table 1), and one with higher efficiency of gene repression was used to suppress the given gene in PU.1-induced MG in mhCOs (Supplementary Fig. 8d). The expression of target genes was dramatically down-regulated in mhCOs by the given gRNA (Supplementary Fig. 8b). We assessed whether the suppression of the AD-associated genes affected the generation of mhCOs. Regardless of the repression of AD-associated genes, mhCOs showed a similar expression of the microglia markers *Tmem119* and *Iba1* (Supplementary Fig. 8e) and presented with a similar number CD11b^+^ microglia-like cells (Fig. 5c), suggesting a successful formation of cortical organoids with microglia. Even though the suppression of AD-associated genes in mhCOs did not alter the density of CD11b^+^ microglia like-cells, the distribution of microglia-like cells in mhCOs is perturbed (Fig. 5c). For instance, in line with the previous study^53^, TREM2 suppression in microglia-like cells could limit their ability to cluster around the Aβ peptides. Thus, our results suggest that downregulation of AD-associated genes in immune cells does not affect microglia formation but their functions in mhCOs.

Next, we examined how suppressing the AD-associated genes in PU.1-induced MG affects the mhCOs when treated with Aβ. Aβ_1-42_-oligo treatment resulted in a dramatic morphological change, particularly on the surface of *Trem2*- or S*orl1*-suppressed mhCOs. However, the suppression of *Cd33* did not have any impact (Fig. 5d). Importantly, a dramatic increase in cell death was observed in TREM2- or SORL1-suppressed mhCOs to an extent as control hCOs treated with Aβ_1-42_-oligo, characterized by cleaved Caspase-3 and TUNEL staining (Fig. 5e and Supplementary Fig. 8f). The suppression of *Cd33* did not affect the microglial immune response to Aβ (Fig. 5e and Supplementary Fig. 8f). In line with previous mice studies^54,55^, CD33 acts upstream of TREM2 and inhibits the Aβ oligo uptake in microglia. Moreover, we further examined the dysregulation of cholesterol metabolism via the *Sorl1* gene, as indicated in the CRISPRi screen (Fig. 5a), using *Sorl1*-suppressed mhCOs. Since cholesterol 24-hydroxylase (CYP46A1) takes part in the removal of cholesterol in the brain^56^, staining for CYP46A1 in organoids was performed (Supplementary Fig. 9a). Notably, *Sorl1*-suppressed mhCOs demonstrated the significantly reduced CYP46A1 expression compared to hCOs and mhCOs with and without Aβ-treatment (Supplementary Fig. 9a). We lastly examined the cholesterol turnover in mhCO variants. Control and mhCO variants possessed similar total cholesterol levels with and without Aβ treatment (Supplementary Fig. 9b). Interestingly, *Sorl1* suppressed mhCOs exhibited significantly increased free cholesterol levels and decreased cholesteryl esters than other organoids (Supplementary Fig. 9b). Thus*, Sorl1* suppression leads to decreased CYP461 expression, in turn, reduced cholesterol turnover rate, consistent with previous study mice study^57^. Overall, these data suggest that the PU.1-induced microglia-like cells in mhCOs are an excellent model to investigate the function of AD-associated genes in responding to Aβ.

## Discussion

As brain resident myeloid cells, microglia play a critical role in the homeostasis of the brain. The dysregulation of microglia is an underlying mechanism in most human brain diseases, including AD, ASD, and Schizophrenia. However, there has been a significant challenge to develop a human in vitro model to investigate microglia, with the primary microglia^21^ or IPSCs-derived microglia^58–60^ as viable options. Recent studies have revealed that we cannot study microglial functions and the related phenotypes without considering the interaction with other cells in the brain^22,61^. Investigating the molecular and cellular responses in microglia necessitates the production of microglia-like cells in the context of a brain structure. Thus, our strategy developing mhCOs opens an opportunity to examine human microglia function in health and disease state closer to brain microglia.

Human brain organoid technology holds great potential to serve as a platform to model diseases and screen drugs for human brain diseases. However, most brain organoids made thus far lack microglia except the recently reported cerebral organoids, the unguided protocol^17^. Here, we employed PU.1-induction during hCO formation, guided protocol, to differentiate hPSCs towards functional microglia-like cells, generating mhCOs. However, cerebral organoids still acquire mesodermal progenitors since they rely on the intrinsic differentiation of hESCs^13^. Therefore, Ormel and his colleagues modified the unguided protocol by decreasing the neuroectoderm stimulant and delaying Matrigel coating. Hence, these modifications lead to the production of mesodermal progenitors and following differentiation of microglia in organoids^17^. While they noted innately developed microglia within cerebral organoids, variability of microglia numbers could become an issue.

In contrast, our mhCOs offer a platform for developing a tunable number of microglia within organoids and studying the microglia-specific function in vitro by genetic modification at either microglia alone or all cells in hCOs. We utilized this feature to determine the role of AD-associated genes in microglia in responding to Aβ by applying pooled CRISPRi screening. Recently, microglia or erythromyeloid progenitors derived from induced PSCs were co-cultured with cortical and cerebral organoids, respectively^14,16,62,63^. However, these protocols require the addition of expensive cytokines and growth factors during the differentiation of immune cells or progenitors separately in time-consuming protocols. In a more recent study, primary microglia from cortical tissues were isolated and combined with cortical organoids without the addition of any microglia survival chemical to investigate the role of microglia on early neurogenesis^15^. This protocol requires the access of primary prenatal human tissue and could be suffered from different developmental stages of neurons from organoids and isolated microglia from primary tissue. On the other hand, mhCOs do not require the steps to differentiate microglia using cytokines and growth factors from hESCs for incorporating them in organoids. Moreover, mhCOs carry microglia-like cells parenchyma and the surface of organoids, closely producing the location and behavior of microglia in the human brain.

There are limitations even though mhCO offers a unique platform to investigate microglia-associated diseases via gene-editing in microglia. One is that the distribution of microglia varies within each mhCOs. Second, not all PU.1 over-expressed cells in mhCOs converted into microglia clusters (Fig. 2a), indicating either inefficient conversion or environmental influence shaping the final cell identity. Thus, generating a different type of organoids with PU.1 expressing cells may lead to myeloid cells with a distinct identity and maturation efficiency. Lastly, mhCOs do not possess an endogenous vessel system and thus contain unhealthy cells at late stages of development. This internal inflammation and lack of cell-type complexity may lead to the premature activation of microglia and developmental maturation different from fetal microglia. Nonetheless, mhCOs open up new avenues to probe the function of microglia readily in human brain organoids for various human brain disorders and brain development.

## Methods

This study complies with all relevant ethical regulations approved by Yale Medical School. All experiments involving hESCs were approved by the Yale Embryonic Stem Cell Research Oversight Committee (ESCRO). All animal experiments described in this study were approved by the Institutional Animal Care & Use Committee (IACUC) of Yale University. The human brain tissues assessed in this study were obtained from the Yale Pathology based on Yale Human Investigation Committee at Yale University. It has been approved for use with a waiver of consent.

### Animals

The Rag2−/− GammaC−/− mice were purchased from Jackson Laboratories. Animals were housed at room temperature (68–79°F) and 40–60% humidity.

### hESCs culture

HES-3 NKX2-1^GFP/w^, BC4, and BC61 hESCs were cultured on Matrigel (BD Biosciences) coated cell culture dishes with mTeSR1 media (Stem Cell Technologies). hESCs were passaged every week by treatment with Dispase (0.83 U/ml, Stem Cell Technologies).

### Generation of BC61 hESCs

As we described previously^27^, we first generated a cassette containing doxycycline-inducible *PU.1-IRES-eGFP* and rTTA and introduced it into the AAVS1 locus of HES3. Briefly, 2 million HES3 cells were electroporated with 8 µg donor plasmid, 1 µg AAVS1 TALEN-L, and 1 µg AAVS1 TALEN-R by using the Amaxa Nucleofector device (AAB-1001, Lonza) and seeded in mTeSR1 plus Y27632 (10 µM). After 3 days, G-418 (Thermo Fisher Scientific) was applied for 7 days (400 µg/ml for the first 3 days and 300 µg/ml for the next 4 days) to obtain stable colonies. Finally, a single isogenic colony was picked and expanded for quality control analysis.

### Generation of human cortical organoids (hCOs) with PU.1 induction

To generate microglia containing cortical organoids, we first generate lentivirus containing PU.1. Firstly, *Pu.1* gene is amplified from pINDUCER-21-SPI1 (addgene #97039) using primers forward: 5′-TTT GGATCCAAGGCCCACCATGGAAGGGT-3′ and reverse: 5′-TTT GTTTAAACTCATTACTAAGCGTAGTCTG-3′ and cloned into pTet-IRES-eGFP (addgene #64238) with PmeI and BamHI. The lentivirus containing inducible *Pu.1* was transduced for 7 days without dox. As described earlier^27^, we generated hCOs by mixing 10% PU.1-infected BC4 and 90% non-infected parental HES3 hESCs. Briefly, after counting the cells with ADAM-CellT (NanoEntek), total 9000 cells, composed of 900 PU.1-infected and 8100 cells HES3, were plated into a well of U-bottom ultra-low-attachment 96-well plate in neural induction medium (DMEM-F12, 15% (v/v) KSR, 5% (v/v) heat-inactivated FBS (Life Technologies),1% (v/v) Glutamax, 1% (v/v) MEM-NEAA, 100 µM β-Mercaptoethanol) supplemented with 10 µM SB-431542, 100 nM LDN-193189, 2 µM XAV-939 and 50 µM Y27632. Basal activation of *Pu.1* was started on day 2 by adding 0.5 µM dox, and FBS and Y27632 were removed from day 2 and 4, respectively. The medium was replenished every other day until day 10, where organoids were transferred to the ultra-low-attachment six-well plate. The organoids were cultured in spinning hCO medium with minus vitamin A (1:1 mixture of DMEM-F12 and Neurobasal media, 0.5% (v/v) N2 supplement, 1% (v/v) B27 supplement without vitamin A, 0.5% (v/v) MEM-NEAA, 1% (v/v) Glutamax, 50 µM β-Mercaptoethanol, 1% (v/v) Penicillin/Streptomycin and 0.025% Insulin). The medium was replenished every other day until day 18, where media was switched to the hCO medium with vitamin A (the same composition as described above except B27 with vitamin A) supplemented with 20 ng/ml BDNF and 200 µM ascorbic acid. The medium was changed every 4 days after day 18. Activation of *Pu.1* was performed beginning on day 18 by adding 2 µM dox continuously in the medium.

### Human tissue

Human tissue was obtained under a protocol approved by the Yale Human Investigation Committee at Yale University. The tissue was immediately placed in an RPMI medium and processed within 10 h of collection. Briefly, 70-year and 77-year-old male brain tissues were cut into 1-mm sections and cultured in hCO culture media in the presence and absence of Aβ_1-42_-oligo for 72 h. Then, samples were collected and processed for analysis.

### Live imaging of mhCOs types

Live images were captured in mhCOs at day 30 and day 70 and mhCO at day 60 to visualize the eGFP-expressing cells and their morphologies. The Leica TCS SP5 confocal microscope, equipped with a controlled cell chamber possessing 37 °C temperature and 5% CO_2_, was used to generate *z* stack images. 3D reconstruction of images was attained by using Leica Las-X software.

### Scanning electron microscopy

The cultured organoids day 75 were fixed in 2.5% glutaraldehyde solution for 30 min. Then, the organoids were applied to a sequential dehydration series of 50%, 75%, 90%, and 100% ethanol. Finally, the surfaces of the hCOs were sputter-coated with gold and imaged by FE-SEM (Model S-4700, Hitachi, Canada).

### Immunofluorescence staining

For organoids, residual media was removed by washing with PBS. As described earlier^27^, all hCOs were fixed in 4% paraformaldehyde (PFA) at 4 °C overnight. After washing with PBS three times, they were incubated in 30% sucrose solution for 2 days at 4 °C. Next, organoids were embedded in O.C.T. in base molds on dry ice and sectioned for 40-μm. The organoid blocks were further stored at −80 °C. After sections were dried, they were incubated with 0.1% Triton-100 for 15 min and further blocked with 3% bovine serum albumin (BSA) for 2 h at RT. Then, the primary antibody, diluted in 3% BSA, incubation is performed at 4 °C overnight. After washing with PBS, organoids were incubated with Alexa Fluor dyes (1:1000) for 1 h and following nuclei staining with DAPI (1:1000) for 10 min at RT. Finally, slides were mounted with ProLong Gold Antifade Reagent, and images were taken with Leica TCS SP5 confocal microscope. The tunnel assay (C100247, Invitrogen) was performed to detect apoptotic cells following the manufacturer’s protocol. A list of antibodies is presented in Supplementary Table 2.

### Whole-mount immunostaining of organoids

We performed whole-mount immunostaining followed by confocal microscopy to examine the localization and organization of Aβ deposition and microglia localization within the cortical organoids. As we described earlier^27^, whole-mount immunostaining of organoids was applied. Briefly, organoids were fixed overnight in 4% paraformaldehyde (PFA) at 4 °C. After extensive washing of the organoids with PBS around 3–5 h, the organoids were blocked overnight at RT in 0.5% BSA and 0.125% Triton-100 in PBS. Organoids were incubated in primary antibodies (anti-IBA1 1:100, anti-MAP2 1:200, anti-PSD95 1:200, and anti-Aβ 1:100) and diluted in 0.5% BSA and 0.125% Triton-100 in PBS for 2 days at 4 °C. Unbound antibodies were removed via washing with PBS for one day at RT. Then, organoids were incubated with Alexa Fluor Dyes (1:500) for 4 h following nuclei staining with DAPI (1:1000) for 2 h. The organoids were cleared by applying to a sequential dehydration series of 30%, 50%, 70%, and 99% 1-propanol (diluted in PBS pH adjusted to 9.5 via triethylamine) for 4 h at 4 °C. After dehydration, the organoids were incubated with ethyl cinnamate for 1 h at RT in light-protected and air-sealed tubes. The images were taken with a Leica TCS SP5 confocal microscope.

### Single-molecule RNA fluorescent in situ hybridization

For sample preparation, whole organoids at day 90 were frozen in Tissue-Tek Cryo-OCT compound (Fisher 811 Scientific) on dry ice and stored at −80 °C until further use. Organoids were sectioned at a thickness of 20 μm, and RNAs were detected by RNAscope (Advanced Cell Diagnostics) based on the manufacturer’s guidelines using the probes for human *Fos* (check the cat. no. 319901) and *Npas4* (check the cat. no. 501121-c2). All processed samples were imaged using an Olympus VS120 slide scanner and analyzed in ImageJ.

### Viral labeling and calcium imaging

As described previously^26^, organoids were transferred to a 96-well plate for viral infection. After AAV. Syn. GCAMP6s.WPRE.SV40 (Addgene, 100843^64^) and AAV5.hSyn. eGFP (Addgene, 50465, was a gift from Bryan Roth) separate incubation in 300 μl neural media for 24 h, organoids were transferred to a six-well plate in a fresh medium. After 10–15 days of virus transduction, the intact organoids were used for calcium and structural imaging. Time-lapse images were taken with Leica TCS SP8 confocal microscope at a speed of 1 s/frame. Tracings of single-cell calcium surges were determined by measuring the region of interest and mean of interest fluorescence intensities using Fiji software^65^. The change in calcium concentration is calculated as follows Δ*F*/*F* = [(*F*(*t*)−*F*0)/*F*0), where *F*0 is calculated as the average of portions without calcium events.

### Real-time quantitative PCR (qPCR)

Total RNA was isolated from the organoids via RNeasy Mini Kit (Qiagen). 1 µg RNA was converted to cDNA using iScript Select cDNA Synthesis Kit. To quantify gene expression, qPCR was carried out on the CFX96 Real-Time PCR system (Biorad) using the SsoFast EvaGreen Supermix (Biorad). The PCR conditions were: 95 °C for 15 min, followed by 40 two-step cycles at 94 °C for 10 s and 60 °C for 45 s. A list of primers used in this study is presented in Supplementary Table 3.

### Library preparation for scRNA-seq

Cortical organoids were randomly collected from three different culture dishes for each time point (Day 90: hCOs and mhCOs with and without Aβ treated a total of 32 organoids pooled; day 75: totally 8 mhCOs pooled together to sort GFP^+^ and GFP^−^ cells). Organoid dissociation, cDNA preparation, and sequencing were performed as we described previously^29^. Briefly, organoids were dissociated using the papain according to the manufacturer’s instructions. After washing once with HBSS, organoids were dissected into small pieces in oxygenated papain solution (all solutions used were oxygenated with 95% O2:5% CO_2_ for around 5 min). Dissected tissue was oxygenated for 5 min and incubated in a 37 °C water bath for 1 h, with gentle shaking every 10 min. After gentle trituration, papain was inactivated in albumin-ovomucoid inhibitor, and single cells were suspended in 1% BSA/PBS supplemented with 10 μM Y27632. Then, cells were stained with propidium iodide (PI) for 15 min on ice, sorted out, and re-suspended at 128 cells/μl. cDNA libraries were generated with the Single Cell 3′ v3 Reagent Kits according to the manufacturer’s instructions. After barcoded full-length cDNA from poly-adenylated mRNA generated, the libraries were then size-selected, and R2, P5, P7 sequences were added to each selected cDNA during end repair and adaptor ligation. After Illumina bridge amplification of the cDNA, each library was sequenced using the Illumina Novaseq S4 2×150 bp in Rapid Run Mode.

### Data processing of scRNA-seq

scRNA-seq reads were aligned to hg19 human genome and counted with Ensembl genes by count function of CellRanger (v3.0.2) with default parameters. All libraries were merged with the normalization to the same sequencing depth by aggr function of CellRanger with default parameters. Before processing scRNA-seq analysis, we confirmed the low doublet frequency of our scRNA-seq libraries (0.82 ± 0.28%) by counting cells expressing both TBR1 and GFAP, which are usually exclusively expressed in cortical neurons and astrocytes, respectively^26,29^.

The batch effect and intrinsic technical effect were minimized by Seurat (v3.0.2)^66^. Briefly, the raw UMI count was normalized to the total UMI count in each library. Highly variable genes were then identified by variance stabilizing transformation with 0.3 loess span and automatic setting of the clip.max value. Top 2500 variable genes were used to identify cell pairs anchoring different scRNA-seq libraries using 20 dimensions of canonical correlation analysis. All scRNA-seq libraries used in this study were integrated into a shared space using the anchor cells. After scaling gene expression values across all integrated cells, we performed dimensional reduction using principal component analysis (PCA). For the visualization, we further projected single cells into two-dimensional UMAP space from 1st and 30th PCs. Graph-based clustering was then implemented with the shared nearest neighbor method from 1st and 20th PCs and a 0.8 resolution value. Differentially expressed genes (DEGs) in each cluster were identified with more than 1.25-fold change and *p* < 0.05 by a two-sided unpaired *T*-test. Gene Ontology analysis was performed on the DEGs by the GOstats Bioconductor package (v2.46.0). False discovery rate was adjusted by p.adjust function in R with “method = BH” parameter.

Cell types were then assigned systematically with unique markers and Gene Ontology enrichment and validated by the reference transcriptome of human brain cell types^30^. First, we isolated neuronal clusters with an expression of neuronal growth cone markers (*Stmn*, *Gap43,* and *Dcx*). Excitatory cortical neuron (CN) and interneuron clusters (IN) were then categorized by their unique markers (*Slc17a6* for CN and *Slc32a1* and *Gad2* for IN). Neuronal clusters without these markers were assigned as non-committed neurons (Neuron).

Non-neuronal clusters were also separated with early neurogenesis markers (VIM, HES1, or SOX2). Clusters expressing or lacking both the growth cone and the early markers were assigned as intermediate (Inter). Neuronal progenitor cell clusters (NPCs) were categorized by high expression of genes related to “mitotic nuclear division (GO:0007067)”. As shown previously^29^, several non-neuronal clusters were characterized by “cilium assembly (GO:0042384)” and “response to BMP (GO:0071772)” and called as a cilia-bearing cell (CBC) and BMP responsible cell (BRC), respectively. Ten non-neuronal clusters displayed high expression of “gliogenesis (GO:0042063)”. In particular, eight out of the glia clusters also highly expressed genes related to “astrocyte differentiation (GO:0048708)” and were assigned as astrocyte clusters (AS). One cluster was annotated as unfolded protein response-related cell (UPRC) with significant enrichment of “endoplasmic reticulum unfolded protein response (GO:0030968)”.

Six non-neuronal clusters were predominantly generated from PU.1-induced cortical organoids. In particular, one of them was characterized by microglia-specific markers (*SPI1* (*PU.1*), *AIF1*, *CSF1R*, *C1QA,* and *C1QB*) and significant enrichment of “leukocyte mediated immunity (GO:0002443)”. Three clusters highly expressed biglycan (BGN) and decorin (DCN) and were annotated as the proteoglycan-expressing cell (PGC). Two clusters were characterized by unique expression of microglia progenitor A1 (*TREM1* and *NFKB1*) and A2 markers (*CXCL1* and *MYB*) as MGPA1 and MGPA2, respectively. One cluster was predominantly generated from Aβ-treated hCOs and characterized as mesodermal markers (*MYL1* and *MYH3*). The rest cluster showed no significant enrichment of GO terms and was called an unassigned cluster (UN).

The reference transcriptome of the fetal and adult brain was downloaded from NCBI Short Read Archive (SRP057196)^30^. Gene signatures for each cell type were obtained as described previously^26^. In each cell, genes were ranked by relative expression to an average of all cells. Gene Set Enrichment Analysis (GSEA) was conducted by GSEAPY software (v0.9.3) with options “–max-size 50000–min-size 0 -n 1000” to the pre-sorted genes.

In each library, we set 15 cells (~0.15%) to define the “generation” of each cell type. With this threshold count, non-treated hCOs did not generate MG (3 cells), MGPA2 (4 cells), MGPA1 (4 cells), and ME (0 cells). MG (12 cells) was also not generated from Aβ-treated hCOs. Both non-treated and Aβ-treated mhCOs did not generate ME (4 and 2 cells), respectively.

Global gene expression comparison between different organoids was performed using all cells in the scRNA-seq dataset. Differential expression was defined by *p* < 1e−50 with a two-sided *T*-test. GO analysis was performed by GOstats as described above.

Single-cell RNA-seq datasets of in vivo human brains from AD patients and healthy donors were downloaded from NCBI Gene Expression Omnibus (GEO) (GSE138852)^39^. The raw sequence reads were processed as described above and merged with our scRNA-seq datasets by CellRanger aggr function. As described above, the UMI count matrix was normalized and projected into the shared UMAP space with Seurat. The annotation of the human brain samples was determined as that of the nearest cells from the organoid samples by calculating the Euclidian distance from 1st to 30th PCs.

We also downloaded the UMI count matrix for droplet-based scRNA-seq of the human fetal brain from UCSC Cell Browser (https://cells.ucsc.edu/?ds=organoidreportcard)^31^. The human fetal brain single-cell transcriptome was integrated with our mhCO scRNA-seq by Seurat as described above. Single-cell transcriptome data from human fetal microglia was obtained from NCBI GEO (GSE141862)^35^ and integrated with cells in the MG cluster from our scRNA-seq. The developmental trajectory was then constructed from the normalized expression values using Monocle (v2.99.3) (Cao et al., 2019). Briefly, we drew a principal tree by the DDRTree algorithm and grouped cells by Louvain clustering with 20 nearest neighbors. The developmental stage (Pseudotime) was then estimated by selecting a cluster that was mainly composed of GW9 microglia. Pearson correlation coefficient between pseudotime and the expression level was calculated by cor function in R for each gene. Gene Ontology analysis was implemented by GOstats for genes with more than 0.2 correlation coefficient.

scRNA-seq for GFP^+^ and GFP^−^ populations were preprocessed by cellranger (v.3.0.2) and Seurat (v3.0.2), as described above. After mapping and quality control, the developmental trajectory was constructed from single cells from GFP^+^ and GFP^−^ populations using DDRTree algorithm with 10 max components in Monocle (v2.99.3)^67^. Trajectory branches were assigned to cell types with their unique markers. To measure the efficiency of MG lineage commitment, we divided cells with GFP and PU.1-derived reads into 100-cells bin and counted the number of cells in MG branch in each bin.

Gene expression profiles of microglia precursor (MP) and differentiated microglia (dMG) were obtained from NCBI GEO (GSE139194)^32^. GSEA of gene signatures for MG, MGPA1 and MGPA2 clusters between dMG and MP was implemented by GSEA software (v2.2.2) with 1000 permutations to gene set, no dataset collapse, and weighted enrichment statistic^68^.

Bulk RNA-seq datasets for AD patient brains in each Braak stage were downloaded from NCBI GEO (GSE110731)^45^. The reads were mapped to hg19 human genome by Tophat2 (v2.2.1) with default parameters^69^. The gene expression value was estimated by Cufflinks (v1.2.0) with RefSeq gene reference annotation^70^. The stage-specific genes were identified with more than 1.25-fold change and *p* < 0.05 by two-sided *T*-test. The statistical enrichment of the stage-specific genes was evaluated by GSEA software (v2.2.2) as described above.

Ferroptosis-induced gene sets were obtained from gene expression profiles in withaferin A (WA)-treated cells (GSE112384)^71^. Up-regulated genes (log2(fold change) > 0.1 and *p* < 0.05 by two-sided *T* test in both IMR32 and SK-N-SH cell lines) in WA-treated samples to control and with a non-treated sample were used as “ferroptosis genes”.

### Data processing of CROP-seq

CROP-seq reads were first aligned to gRNA plasmid library sequences by Bowtie2 (v2.3.0)^72^. Then, CROP-seq datasets were merged with scRNA-seq datasets and processed by CellRanger and Seurat software as described above to annotate of cell types. The annotation of individual cells from the CROP-seq dataset was labeled as the nearest cells from scRNA-seq by calculating Euclidean distance from first and 20th PCs. Differentially expressed genes between Aβ_1-42_ and non-treatment mhCOs were identified in each knockdown with a 1.25-fold change and *p* < 0.05 by two-sided paired *T*-test.

### Quantification of cholesterol levels

Cellular cholesterol levels were extracted from organoid tissue. Briefly,10 mg tissues were dissolved with 200 μl of chloroform:isopropanol:NP40 (7:11:0.1) in a micro-homogenizer. After 10 min of room temperature incubation, tissue extract was spun at 15,000×*g* for 10 min. Next, the liquid was transferred to a glass tube and air-dried for 30 min to remove the organoid solution. The dried lipids were dissolved with 250 μl of cholesterol assay buffer, vortexing until homogenous. Then, the cholesterol levels were measured from 50 μl of the extracted sample using the Amplex Red cholesterol assay kit (Invitrogen). Briefly, cholesterol oxidase converts cholesterol to hydrogen peroxide and ketones. The hydrogen peroxide reacts with Amplex Red reagent (10-acetyl-3,7-dihydroxyphenoxazine), producing resorufin, fluorescence monitored excitation/emission couple of 545/590 nm on a PerkinElmer plate reader.

### Transplantation of PU.1-induced microglia into mouse brain

PU.1-derived microglia were transplanted into mice brains as previously described^73^. Briefly, PU.1 was induced for 5 days in BC4 hESCs to generate microglia-like cells. Then, these cells dissociated and suspended in PBS (100,000 cells/μl). 10 µM Aβ_oligo was prepared by dissolving Aβ in Tris-EDTA buffer (50 mM Tris and 1 mM EDTA, pH 7.5). After mice (postnatal day 4) were anesthetized by hypothermia, 100 K microglia-like cell suspension and 5 µl Aβ_oligo (10 µM) were bilaterally injected into mice brain (4 animals, 2 male and 2 female). Then, mice were recovered on a heating pad at 37 °C. After 21 days of microglia transplantation, mice were perfused with PBS and 4% PFA. Then, the explanted brain tissues were further fixed and sliced for immunofluorescence staining.

### Statistics

Data are presented as mean ± SEM. The paired or unpaired two-tail *t*-test (GraphPad Prism software version 8.2.0), hypergeometric test adjusted by Benjamini–Hochberg procedure, and two-sided *t*-test (R version 3.5.0 software) were used to determine the statistical significance. Statistical tests and biological replicates for each experiment are presented in the figure legends.

### Reporting summary

Further information on research design is available in the Nature Research Reporting Summary linked to this article.

## Supplementary information

Supplementary Information

Description of Additional Supplementary Files

Supplementary Movie 1

Supplementary Movie 2

Supplementary Movie 3

Supplementary Movie 4

Supplementary Movie 5

Supplementary Movie 6

Reporting summary

## Data Availability

All relevant data supporting this study are available from the corresponding authors upon request. Source Data is provided within this paper. Single-cell transcriptome data of in vivo human brain from AD patients and healthy donors (GSE138852)^39^, and human fetal microglia are available (GSE141862)^35^ at Gene Expression Omnibus (GEO). Gene expression profiles of microglia precursor (MP) and differentiated microglia (dMG) (GSE139194)^32^, AD patient brains in each Braak stage (GSE110731)^45^, and ferroptosis-induced gene sets (GSE112384)^71^ available at GEO. Single-cell transcriptome data of the current study are available under accession code GSE175722 at GEO. Source data are provided with this paper.

## Source data

Source Data

## References

[1] Paolicelli RC (2011). Synaptic pruning by microglia is necessary for normal brain development. Science.

[2] Schafer DP (2012). Microglia sculpt postnatal neural circuits in an activity and complement-dependent manner. Neuron.

[3] Parkhurst CN (2013). Microglia promote learning-dependent synapse formation through brain-derived neurotrophic factor. Cell.

[4] Salter MW, Stevens B (2017). Microglia emerge as central players in brain disease. Nat. Med..

[5] Voineagu I (2011). Transcriptomic analysis of autistic brain reveals convergent molecular pathology. Nature.

[6] Wang Y (2015). TREM2 lipid sensing sustains the microglial response in an Alzheimer’s disease model. Cell.

[7] Arlotta P (2018). Organoids required! A new path to understanding human brain development and disease. Nat. Methods.

[8] Eiraku M (2008). Self-organized formation of polarized cortical tissues from ESCs and its active manipulation by extrinsic signals. Cell Stem Cell.

[9] Pasca AM (2015). Functional cortical neurons and astrocytes from human pluripotent stem cells in 3D culture. Nat. Methods.

[10] Lancaster MA (2013). Cerebral organoids model human brain development and microcephaly. Nature.

[11] Velasco S (2019). Individual brain organoids reproducibly form cell diversity of the human cerebral cortex. Nature.

[12] Tanaka Y, Cakir B, Xiang Y, Sullivan GJ, Park IH (2020). Synthetic analyses of single-cell transcriptomes from multiple brain organoids and fetal brain. Cell Rep..

[13] Quadrato G (2017). Cell diversity and network dynamics in photosensitive human brain organoids. Nature.

[14] Lin YT (2018). APOE4 causes widespread molecular and cellular alterations associated with Alzheimer’s disease phenotypes in human iPSC-derived brain cell types. Neuron.

[15] Popova, G. et al. Human microglia states are conserved across experimental models and regulate neural stem cell responses in chimeric organoids. *Cell Stem Cell*10.1016/j.stem.2021.08.015 (2021). 10.1016/j.stem.2021.08.015 PMC8642295 34536354

[16] Fagerlund, I. et al. Microglia-like cells promote neuronal functions in cerebral organoids. *Cells* **11**, 124 (2021). 10.3390/cells11010124 PMC8750120 35011686

[17] Ormel PR (2018). Microglia innately develop within cerebral organoids. Nat. Commun..

[18] Kierdorf K (2013). Microglia emerge from erythromyeloid precursors via Pu.1- and Irf8-dependent pathways. Nat. Neurosci..

[19] Feng R (2008). PU.1 and C/EBPalpha/beta convert fibroblasts into macrophage-like cells. Proc. Natl Acad. Sci. USA.

[20] Forsberg M (2010). Efficient reprogramming of adult neural stem cells to monocytes by ectopic expression of a single gene. Proc. Natl Acad. Sci. USA.

[21] Galatro TF (2017). Transcriptomic analysis of purified human cortical microglia reveals age-associated changes. Nat. Neurosci..

[22] Gosselin, D. et al. An environment-dependent transcriptional network specifies human microglia identity. *Science* **356**, 10.1126/science.aal3222 (2017). 10.1126/science.aal3222 PMC5858585 28546318

[23] Perdiguero EG, Geissmann F (2016). The development and maintenance of resident macrophages. Nat. Immunol..

[24] Schulz C (2012). A lineage of myeloid cells independent of Myb and hematopoietic stem cells. Science.

[25] Lawson LJ, Perry VH, Gordon S (1992). Turnover of resident microglia in the normal adult mouse brain. Neuroscience.

[26] Xiang Y (2017). Fusion of regionally specified hPSC-derived organoids models human brain development and interneuron migration. Cell Stem Cell.

[27] Cakir, B. et al. Engineering of human brain organoids with a functional vascular-like system. *Nat. Methods*10.1038/s41592-019-0586-5 (2019). 10.1038/s41592-019-0586-5 PMC6918722 31591580

[28] Guttikonda SR (2021). Fully defined human pluripotent stem cell-derived microglia and tri-culture system model C3 production in Alzheimer’s disease. Nat. Neurosci..

[29] Xiang Y (2019). hESC-derived thalamic organoids form reciprocal projections when fused with cortical organoids. Cell Stem Cell.

[30] Darmanis S (2015). A survey of human brain transcriptome diversity at the single cell level. Proc. Natl Acad. Sci. USA.

[31] Bhaduri A (2020). Cell stress in cortical organoids impairs molecular subtype specification. Nature.

[32] Svoboda DS (2019). Human iPSC-derived microglia assume a primary microglia-like state after transplantation into the neonatal mouse brain. Proc. Natl Acad. Sci. USA.

[33] Pasca SP (2018). The rise of three-dimensional human brain cultures. Nature.

[34] Lancaster MA, Knoblich JA (2014). Organogenesis in a dish: modeling development and disease using organoid technologies. Science.

[35] Kracht L (2020). Human fetal microglia acquire homeostatic immune-sensing properties early in development. Science.

[36] Condello, C., Yuan, P., Schain, A. & Grutzendler, J. Microglia constitute a barrier that prevents neurotoxic protofibrillar A beta 42 hotspots around plaques. *Nat. Commun.* **6**, ARTN 6176 10.1038/ncomms7176 (2015). 10.1038/ncomms7176 PMC4311408 25630253

[37] Bard F (2000). Peripherally administered antibodies against amyloid beta-peptide enter the central nervous system and reduce pathology in a mouse model of Alzheimer disease. Nat. Med..

[38] Keren-Shaul H (2017). A unique microglia type associated with restricting development of Alzheimer’s disease. Cell.

[39] Grubman A (2019). A single-cell atlas of entorhinal cortex from individuals with Alzheimer’s disease reveals cell-type-specific gene expression regulation. Nat. Neurosci..

[40] Barinaga M (1998). Is apoptosis key in Alzheimer’s disease?. Science.

[41] Wyss-Coray T (1997). Amyloidogenic role of cytokine TGF-beta1 in transgenic mice and in Alzheimer’s disease. Nature.

[42] Abdalkader M, Lampinen R, Kanninen KM, Malm TM, Liddell JR (2018). Targeting Nrf2 to suppress ferroptosis and mitochondrial dysfunction in neurodegeneration. Front. Neurosci..

[43] Yang WS (2016). Peroxidation of polyunsaturated fatty acids by lipoxygenases drives ferroptosis. Proc. Natl Acad. Sci. USA.

[44] Malhotra D (2010). Global mapping of binding sites for Nrf2 identifies novel targets in cell survival response through ChIP-Seq profiling and network analysis. Nucleic Acids Res..

[45] Li P (2019). Epigenetic dysregulation of enhancers in neurons is associated with Alzheimer’s disease pathology and cognitive symptoms. Nat. Commun..

[46] Braak H, Alafuzoff I, Arzberger T, Kretzschmar H, Del Tredici K (2006). Staging of Alzheimer disease-associated neurofibrillary pathology using paraffin sections and immunocytochemistry. Acta Neuropathol..

[47] Efthymiou AG, Goate AM (2017). Late onset Alzheimer’s disease genetics implicates microglial pathways in disease risk. Mol. Neurodegener..

[48] Hollingworth P (2011). Common variants at ABCA7, MS4A6A/MS4A4E, EPHA1, CD33 and CD2AP are associated with Alzheimer’s disease. Nat. Genet..

[49] Datlinger P (2017). Pooled CRISPR screening with single-cell transcriptome readout. Nat. Methods.

[50] Harold D (2009). Genome-wide association study identifies variants at CLU and PICALM associated with Alzheimer’s disease. Nat. Genet..

[51] Lambert JC (2013). Meta-analysis of 74,046 individuals identifies 11 new susceptibility loci for Alzheimer’s disease. Nat. Genet..

[52] Gao Q, Wu G, Lai KWC (2020). Cholesterol modulates the formation of the Aβ ion channel in lipid bilayers. Biochemistry.

[53] Parhizkar S (2019). Loss of TREM2 function increases amyloid seeding but reduces plaque-associated ApoE. Nat. Neurosci..

[54] Griciuc, A. et al. TREM2 acts downstream of CD33 in modulating microglial pathology in Alzheimer’s disease. *Neuron*10.1016/j.neuron.2019.06.010 (2019). 10.1016/j.neuron.2019.06.010 PMC6728215 31301936

[55] Griciuc A (2013). Alzheimer’s disease risk gene CD33 inhibits microglial uptake of amyloid beta. Neuron.

[56] Lund EG, Guileyardo JM, Russell DW (1999). cDNA cloning of cholesterol 24-hydroxylase, a mediator of cholesterol homeostasis in the brain. Proc. Natl Acad. Sci. USA.

[57] Xie C, Lund EG, Turley SD, Russell DW, Dietschy JM (2003). Quantitation of two pathways for cholesterol excretion from the brain in normal mice and mice with neurodegeneration. J. Lipid Res..

[58] Muffat J (2016). Efficient derivation of microglia-like cells from human pluripotent stem cells. Nat. Med..

[59] Abud EM (2017). iPSC-derived human microglia-like cells to study neurological diseases. Neuron.

[60] Pandya H (2017). Differentiation of human and murine induced pluripotent stem cells to microglia-like cells. Nat. Neurosci..

[61] Haenseler W (2017). A highly efficient human pluripotent stem cell microglia model displays a neuronal-co-culture-specific expression profile and inflammatory response. Stem Cell Rep..

[62] Song L (2019). Functionalization of brain region-specific spheroids with isogenic microglia-like cells. Sci. Rep..

[63] Xu R (2021). Developing human pluripotent stem cell-based cerebral organoids with a controllable microglia ratio for modeling brain development and pathology. Stem Cell Rep..

[64] Chen TW (2013). Ultrasensitive fluorescent proteins for imaging neuronal activity. Nature.

[65] Schindelin J (2012). Fiji: an open-source platform for biological-image analysis. Nat. Methods.

[66] Stuart T (2019). Comprehensive integration of single-cell data. Cell.

[67] Cao J (2019). The single-cell transcriptional landscape of mammalian organogenesis. Nature.

[68] Subramanian A (2005). Gene set enrichment analysis: a knowledge-based approach for interpreting genome-wide expression profiles. Proc. Natl Acad. Sci. USA.

[69] Trapnell C, Pachter L, Salzberg SL (2009). TopHat: discovering splice junctions with RNA-Seq. Bioinformatics.

[70] Trapnell C (2010). Transcript assembly and quantification by RNA-Seq reveals unannotated transcripts and isoform switching during cell differentiation. Nat. Biotechnol..

[71] Hassannia B (2018). Nano-targeted induction of dual ferroptotic mechanisms eradicates high-risk neuroblastoma. J. Clin. Investig..

[72] Langmead B, Salzberg SL (2012). Fast gapped-read alignment with Bowtie 2. Nat. Methods.

[73] Mancuso R (2019). Stem-cell-derived human microglia transplanted in mouse brain to study human disease. Nat. Neurosci..

