# Supplementary material for: Expression of the transcription factor PU.1 induces the generation of microglia-like cells in human cortical organoids
Source: Nat Commun. 2022 Jan 20;13:430. doi: 10.1038/s41467-022-28043-y (PMC8776770; doi:10.1038/s41467-022-28043-y)
Supplement: Supplementary file 1 — Supplementary Information [file 41467_2022_28043_MOESM1_ESM.pdf]

Supplementary Fig 1

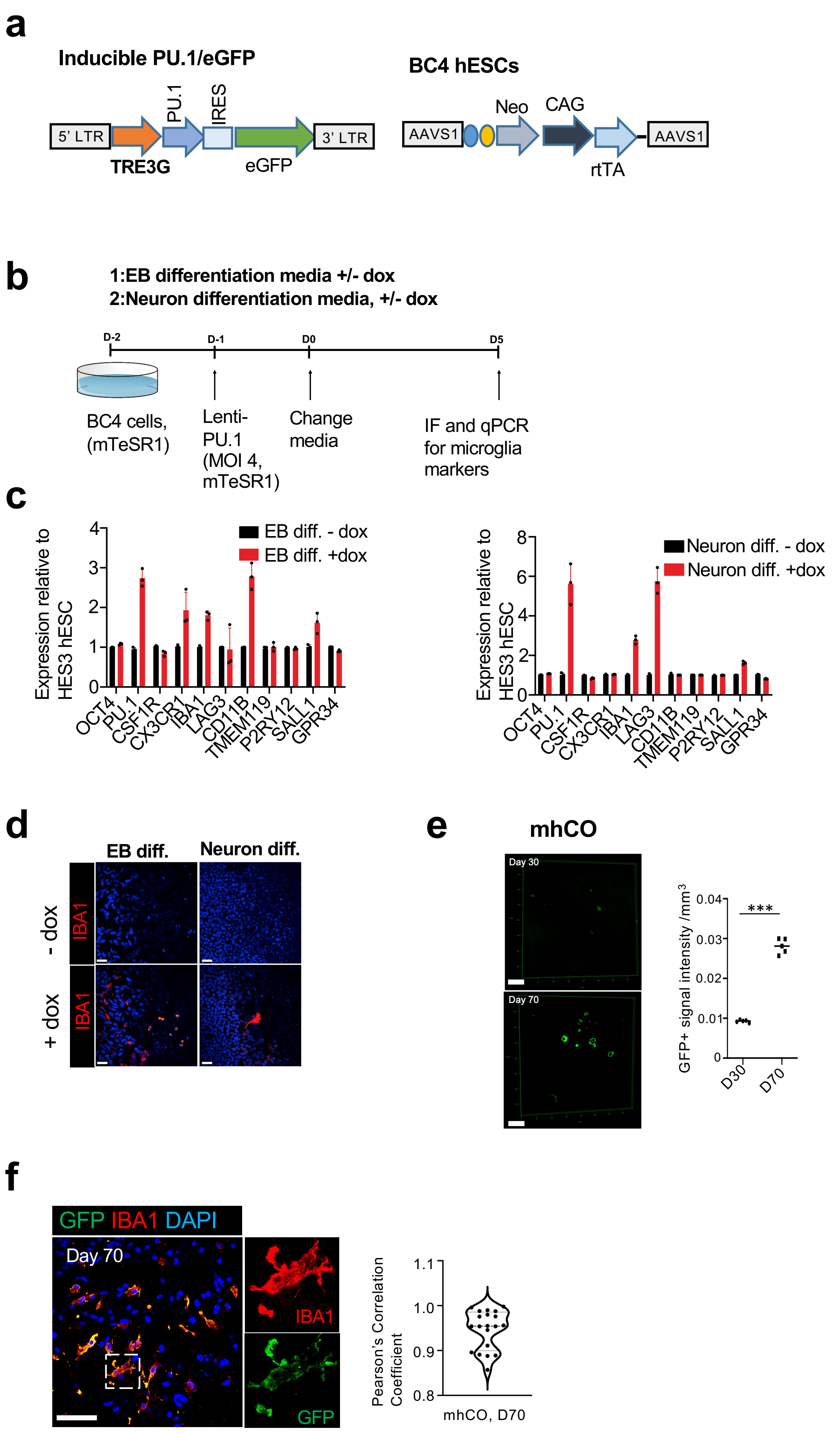

Supplementary Fig. 1. Generation of cortical organoids with microglia-like cells via induction of PU.1 expression. a, Left, a construct of inducible PU.1/eGFP to generate lentivirus. Right, a construct to target the AAVS1 locus to generate rtTA expressing hESC line (BC4). **b,** Depiction of infection of PU.1 (MOI 4) under the embryonic body (EB) or neuron differentiation media for five days. **c,** Expression of microglia-related genes under EB (top) and neuron (bottom) differentiation condition were measured relative to control hESCs and normalized to *β-Actin*. Data represent the mean ± SEM (n=3 three independent differentiation replicates of a hESCs line). **d,** Immunostaining for microglia marker IBA1 in hESCs differentiated without or with PU.1 induction in EB, or neuron differentiation media. Imaging was repeated in samples from three independent differentiation experiments with similar results. **e,** Left, representative images are demonstrating GFP^+ cells expressing *PU.1* that form amoeboid-like structures. Right, quantification of GFP^+ signal intensity per mm^3 in mhCOs at days 30 and 70. Z stack confocal imaging was performed (~ 40-50 μm). Data represent the mean ± SEM (n=5 organoids from three independent differentiation replicates of a hESCs line). Unpaired two-tail t-test was used for comparison (T=21.87, d.f.=8 and ***p<0.000001). **f,** Left, co-immunostaining for GFP and IBA1 in mhCOs at day 70. Right, quantification of co-expression of GFP and IBA1 cells. Data represent the mean ± SEM (n=5, from three independent batches). Bottom, pearson's correlation coefficient of IBA1 with GFP in mhCOs at day 70. The scale bar represents 50 μm in **d**, **e**, and **f**.

Supplementary Fig 2

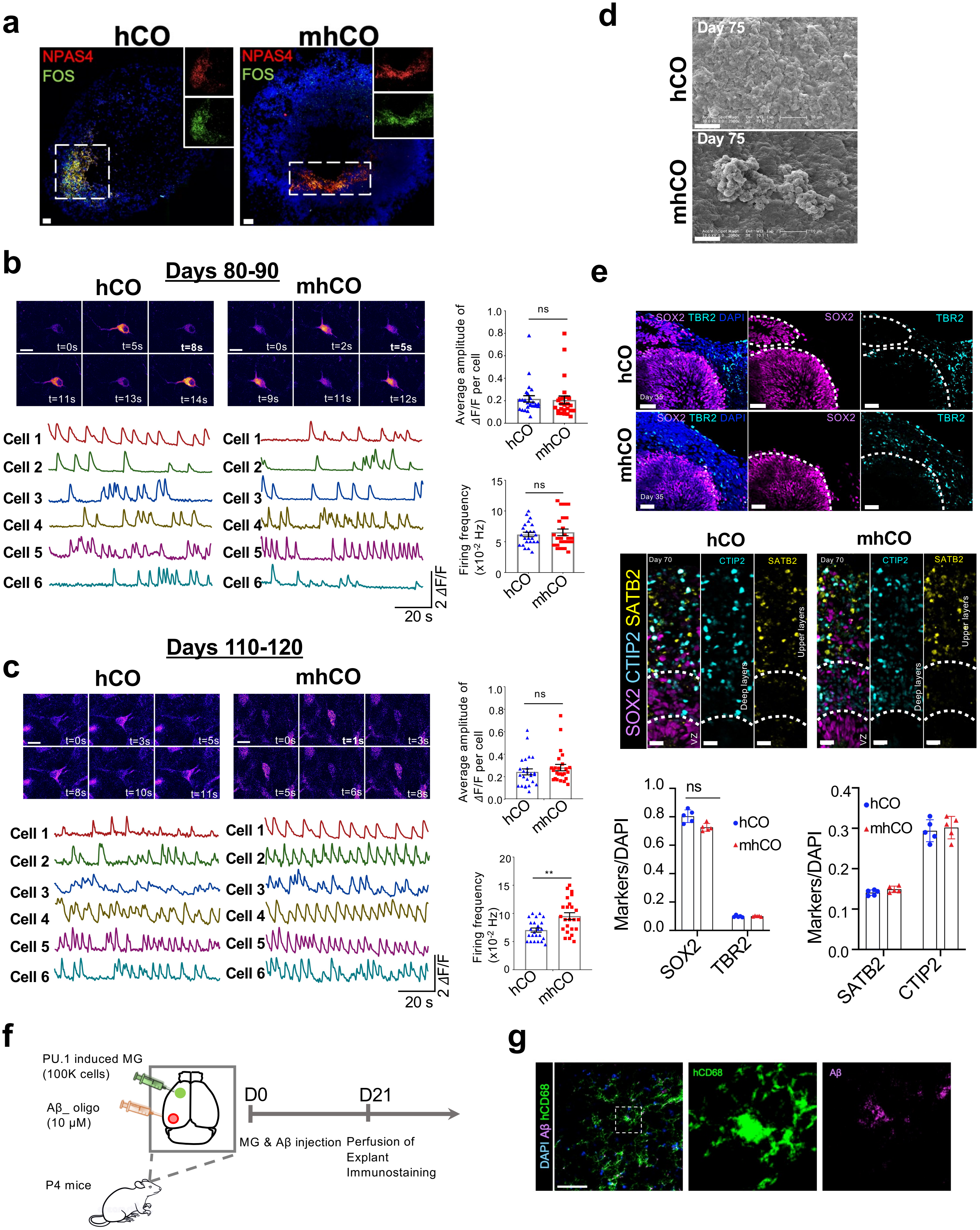

Supplementary Fig. 2. Effects of microglia-like cells in mhCOs and PU.1 induced microglia engraft mouse brain. a, FISH images of organoid sections with NPAS4 and FOS probes. Imaging was repeated in 5 different organoids from one differentiation experiments with similar results. **b-c,** Left, representative images demonstrating calcium transient traces observed from individual neurons of control- and mhCOs at days 70-80 (**b**) and at days 110-120 (**c**). The single-cell tracings of calcium transient were recorded in control and mhCO organoids. Right, the average amplitude of ΔF/F per cell and firing frequency of neurons from hCOs and mhCOs. Error bars denote the mean ± SEM. The unpaired two-tailed t-test was used for all comparisons in **b** and **c** (n=25 neurons from five independent differentiation experiments of a hESCs line in **b** and **c**, **p=0.0012). **d,** SEM image shows the presence of microglia-like cells from mhCO, but not in hCO, at day 75 (2000X magnification). Imaging was repeated in organoids from three independent differentiation experiments with similar results. **e,** Left, co-Immunostaining for SOX2 and TBR2, and SOX2, CTIP2, and SATB2 in 35- and 70-day old control hCOs and mhCOs, respectively. Right, quantification of $SOX2^+$, $TBR2^+$, $SATB^+$ and $CTIP2^+$ cells ratio over $DAPI^+$ cells within organoids. Data are representative images of 5 organoids from three independent experiments. Data indicate the mean ± SEM. The unpaired two-tailed t-test was used for all comparisons. **f,** Depiction of transplantation of PU.1 derived microglia and Aβ_oligo in the immune-deficient mice brain. Green and pink circles, respectively, represent microglia and Aβ_oligo injection sites. **g,** Co-immunostaining for human-specific CD68 and Aβ indicates that successfully engrafted PU.1 induced microglia phagocytoses injected Aβ_oligo. n=4 animals. The scale bar represents 100 μm in **a**, 10 μm in **b**, **d**, and 50 μm in **e**, **g**.

Supplementary Fig 3

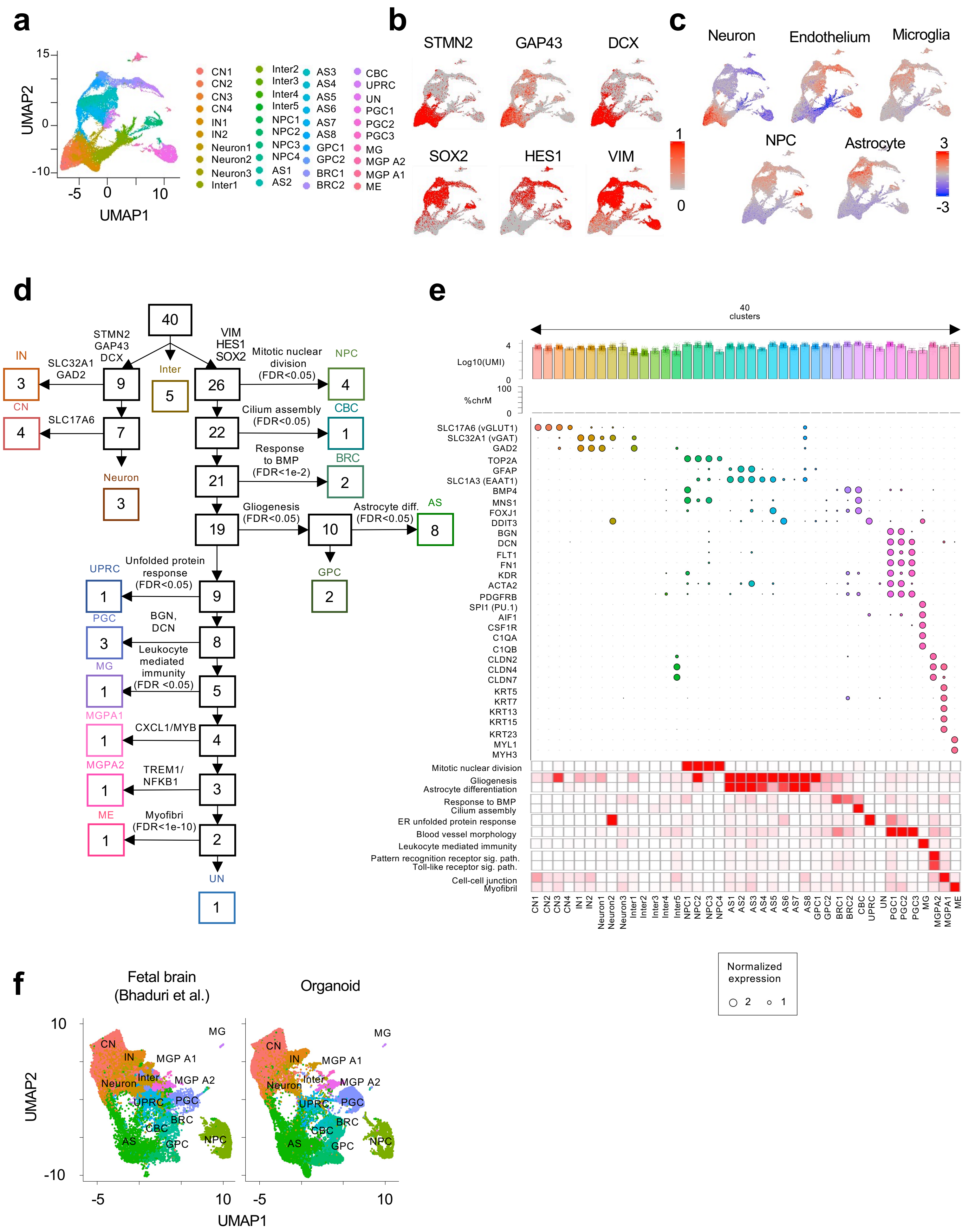

Supplementary Fig. 3. Cluster labeling of single-cell transcriptome analysis.

a, UMAP plot of single cells from non- and Aβ-treated hCOs and mhCOs colored by cell clusters. All 49,867 cells from non- and Aβ-treated hCOs and mhCOs are shown. **b,** Expression patterns of neuronal (*Stmn2*, *Gap43*, and *Dcx*) and early neurogenesis marker (*Sox2*, *Hes1*, and *Vim*). Data depicts results from 49,867 cells from non- and Aβ-treated hCOs and mhCOs. **c,** GSEA of gene signatures for neuron, NPC, endothelium, astrocyte, and microglia. Enrichment and depletion are scaled by –log10(FDR) and shown by red and blue colors, respectively. Data depicts results from 49,867 cells from non- and Aβ-treated hCOs and mhCOs. **d,** Schematic representation of cluster labeling method. ME: mesoderm. **e,** Comparison of average UMI, mitochondrial-derived reads, marker expression, and GO enrichment across 40 clusters (each cluster comprises of 2435, 2042, 1596, 177, 1316, 2658, 2152, 915, 285, 4244, 2813, 316, 585, 371, 187, 583, 885, 218, 3502, 1788, 1595, 623, 785, 2516, 1670, 336, 1770, 1403, 1449, 1401, 1103, 1061, 543, 2349, 609, 144, 198, 939, 225, and 80 (from left to right) biologically independent cells). Data represent the mean ± SEM. **f,** Plot of single cells from mhCOs and human brains in the shared UMAP space colored by cell type assignment [31].

Supplementary Fig 4

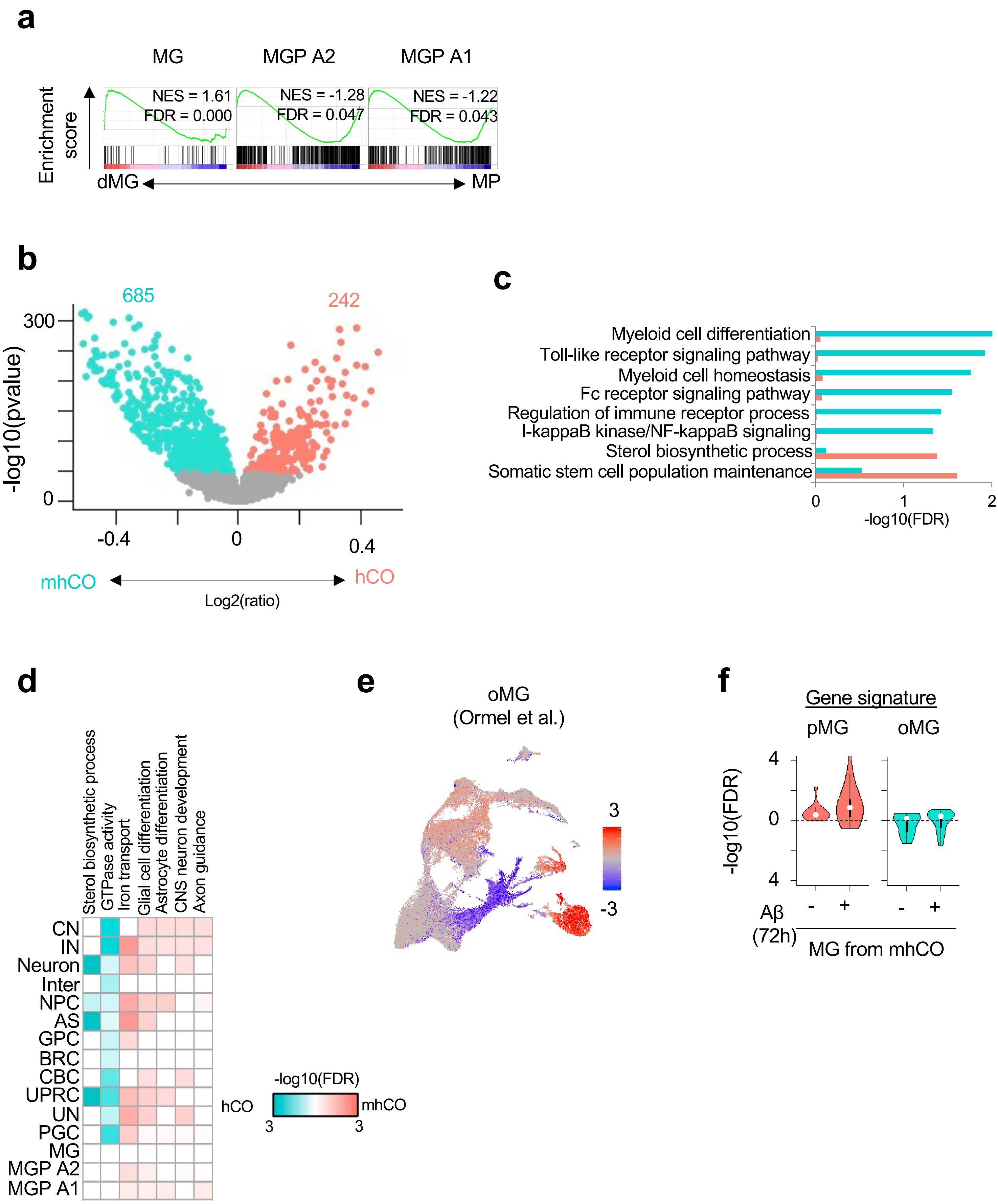

Supplementary Fig. 4. Characterization of cell types in mhCOs using scRNAseq. a, GSEA of gene signatures for MG, MGPA1, and MGPA2 clusters between differentiated microglia (dMG) and microglia precursor (MP). **b,** Volcano plot representing differential gene expression between mhCO and hCO. Differential expression is evaluated by p-value with two-sided T test. 685 up- and 1149 down-regulated genes ($p<1e-50$) are shown by turquoise and salmon color, respectively. The other 31,811 genes are shown by gray color. **c,** GO enrichment of differentially expressed genes in mhCO. **d,** GO analysis for DEGs in each cell type between hCO and mhCO. **e,** GSEA of gene signatures for organoid-grown microglia-like cell (oMG) by Ormel and his colleagues [17]. Enrichment and depletion are scaled by -log10(FDR) and shown as red and blue colors, respectively. **f,** Enrichment of gene signatures for primary microglia (pMG) and oMG by Ormel et al.[17] in our MG cells in mhCOs. Boxes represent 25% and 75% percentile, where white points represent median. Minima and maxima are shown by edges. (n=157 and 26 biologically independent microglia cells in Aβ- and Aβ+ condition, respectively. P-value is 4.70e-2 by two-sided T test).

Supplementary Fig 5

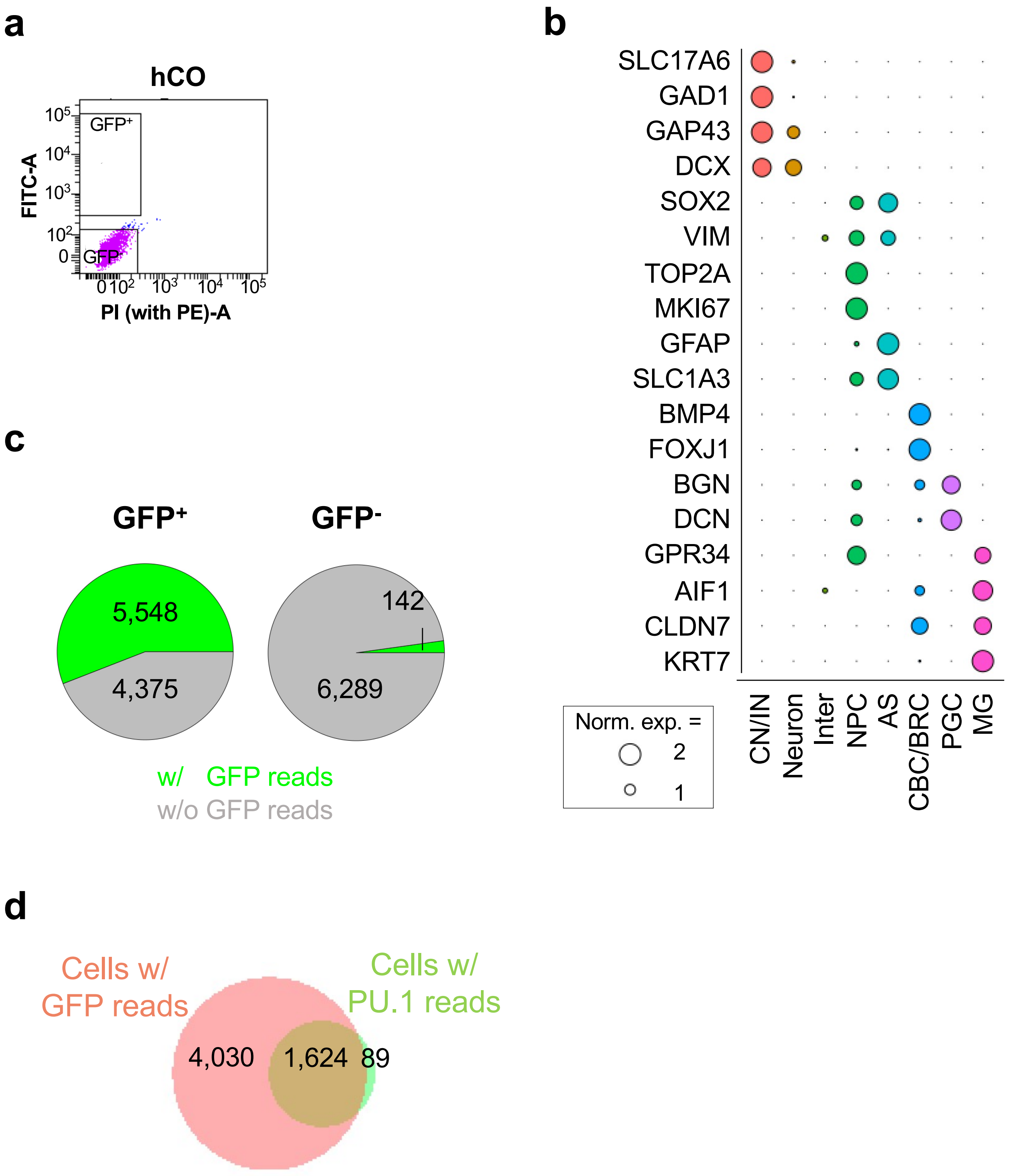

Supplementary Fig. 5. Single-cell transcriptome analysis of GFP+ and GFP- population in the organoid. a, FACS analysis of dissociated hCOs at day 75 shows that GFP^+ cells are not present in hCOs. **b,** Marker expression pattern across branches (n=2036 (CN/IN), 2435 (Neuron), 1913 (Intermediate), 666 (NPC), 3025 (AS), 3107 (CBC/BRC), 1313 (PGC), and 1859 (MG) biologically independent cells). Data represent the mean ± SEM. **c,** The number of cells including GFP-derived reads in GFP^+ and GFP^- libraries. **d,** The number of cells co-expressing GFP and PU.1.

Supplementary Fig 6

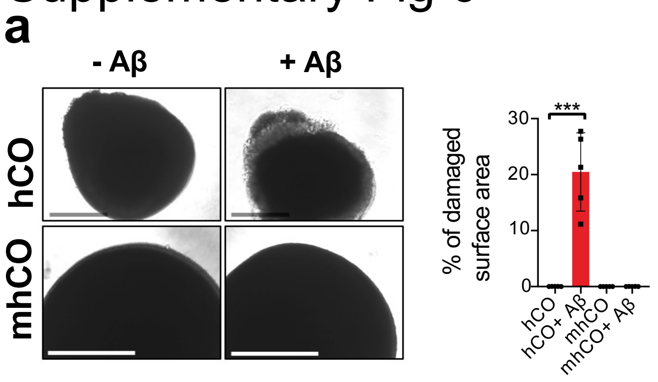

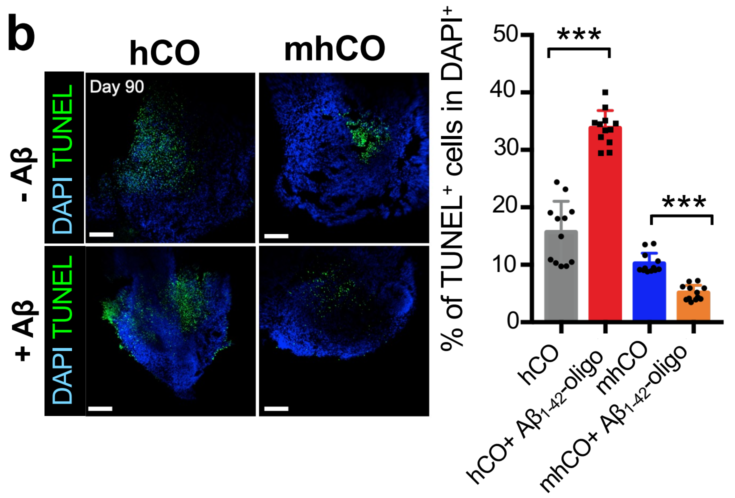

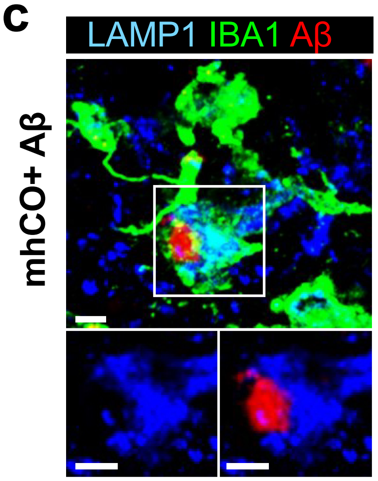

Supplementary Fig. 6. Characterization of Aβ treatment on cortical organoids.

a, Left, representative images are showing the morphology of control hCOs and mhCOs with and without Aβ treatment. Right, quantification of % of surface damage on organoids with and without Aβ treatment. Data are representative images of 5 organoids from three independent experiments. Data represent the mean ± SEM (The unpaired two-tailed t-test was used, T=6.542, d.f.=8, and ***p=0.00018). **b,** Left, TUNEL staining of organoids after 90-day culture. Right, quantification of $TUNEL^{+}/DAPI^{+}$ cells in hCOs and mhCOs at days 90. Data represent the mean ± SEM (n=12 organoids from three independent differentiation replicates of a hESCs line). Unpaired two-tail t-test was used for comparison (T=7.966, d.f.=22, and ***p=0.000000063). **c,** Representative immunostaining of IBA1, LAMP1 and Aβ in hCOs exposed for 72h to Aβ_oligo (n=5, from three independent batches). The scale bar represents 1 mm in **a**, 50 μm in **b**, and 5 μm in **c**.

Supplementary Fig 7

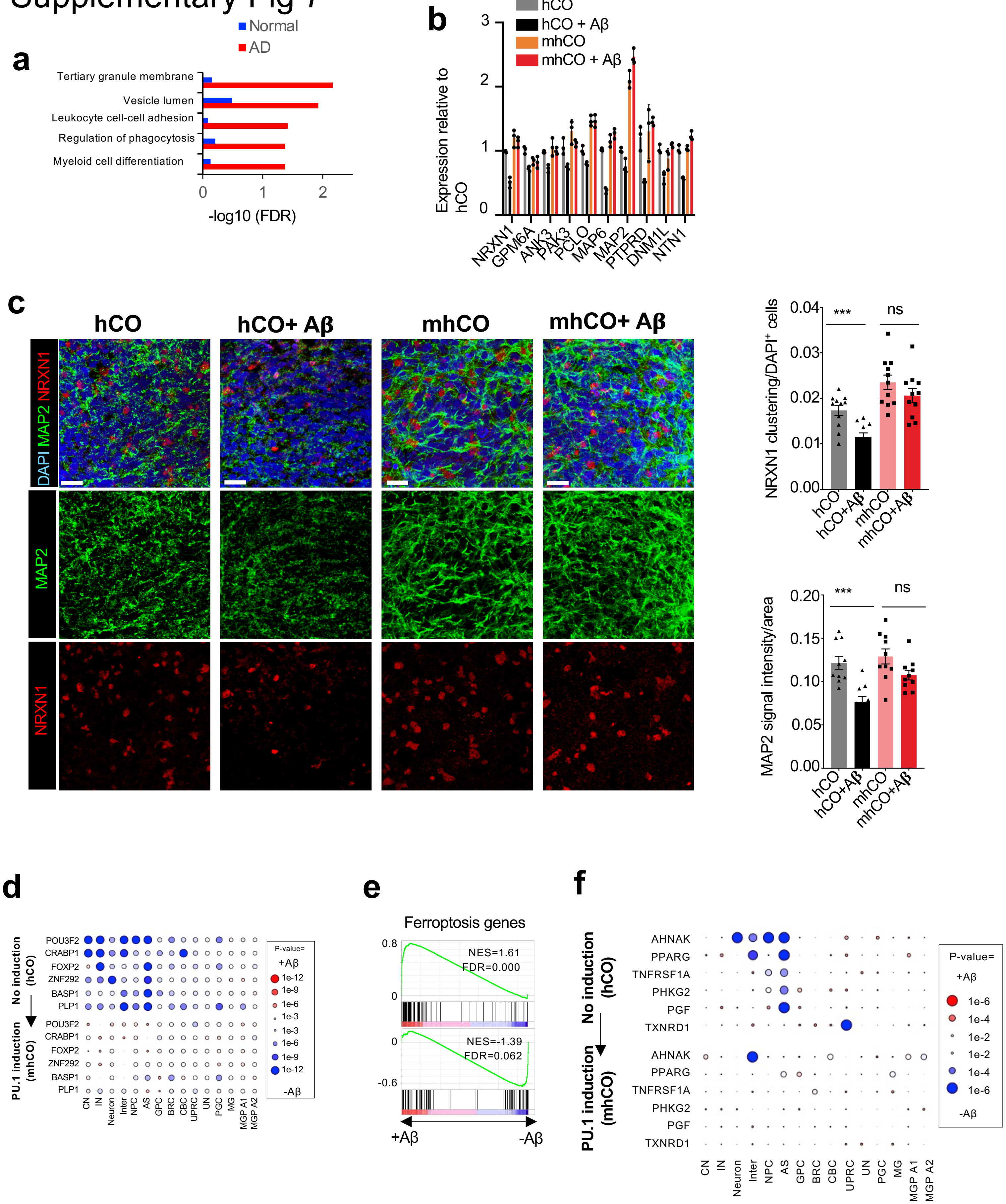

Supplementary Fig. 7. Characterization of Aβ treatment on cortical organoids.

a, GO enrichment of upregulated genes in microglia clusters from AD patient-derived brain compared to healthy donor brain. **b,** Expression of dendrite and synaptic-related genes from control hCOs and mhCOs with and without Aβ treatment. Gene expression was measured relative to control organoids without Aβ treatment and normalized to β-Actin. Data represent the mean ± SEM (n=3, from three independent differentiation replicates of a hESCs line). **c,** Left, immunostaining of control hCOs and mhCOs with and without Aβ treatment for NRXN1 and MAP2. Right, quantification of NRXN1 clustering per DAPI and MAP2 signal intensity per area from organoids with and without Aβ treatment. Error bars denote the mean ± SEM. The unpaired two-tailed t-test was used for all comparisons (n=10 organoids from three independent differentiation replicates of a hESCs line, ***p=0.0002 for NRXN1 and ***p=0.0006 for MAP2). **d,** Differential expression of neuronal and glial differentiation genes with Aβ treatment. Upper and lower panel represents hCO and mhCO datasets, respectively. Differential expression level (-log10(p-value)) was visualized with circle size. Up and down-regulation was scaled from red to blue colors. Two-sided t-test was used for comparison. **e,** Differential expression of neuronal and glial differentiation genes with Aβ treatment. Upper and lower panels represent hCO and mhCO datasets, respectively. Differential expression level (-log10(p-value)) was visualized with circle size. Up- and down-regulation was scaled from red to blue colors. **f,** Differential expression of ferroptosis-related genes with Aβ treatment. Two-sided t-test was used for comparison.

Supplementary Fig 8

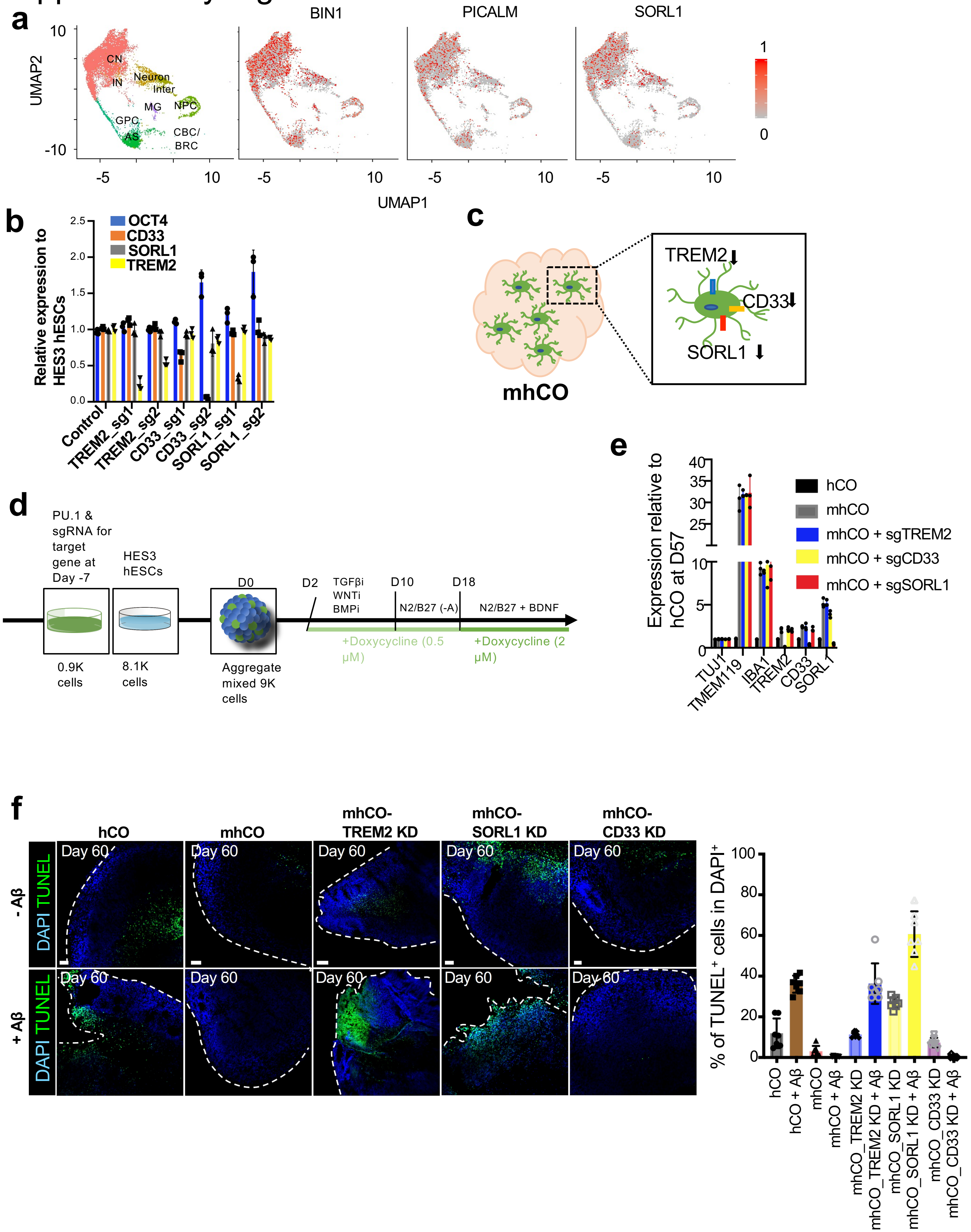

Supplementary Fig. 8. Generation of mhCOs expressing knockdown of AD-associated microglia genes. **a,** UMAP plot of CROP-seq from non- and Aβ-treated mhCOs are colored by cell types. The expression pattern of endocytosis-related genes across cell types is shown by red color. **b,** qPCR analysis of HES3 hESCs containing 2 different gRNAs against the AD-associated microglia genes demonstrated different knockdown efficiency after 6 induction days. UMAP plot of single cells from non- and Aβ-treated hCOs colored by cell type assignment (left) and organoid type (right). Gene expression was measured relative to un-induced HES3 hESCs and normalized to *β-Actin*. Data represent the mean ± SEM (n=3 organoids from three independent differentiation replicates of two hESCs lines). **c,** Depiction of target genes to knock down from mhCOs. **d,** Schematic of the method for generating mhCOs with an AD-associated microglia gene knockdown. Timeline and lentivirus bearing gRNAs of target genes used for cortical organoids are shown. **e,** Expression of microglia-related genes and target genes from mhCO variants at day 57 was measured relative to control hCOs. Data represent the mean ± SEM (n=5, from three independent batches). **f,** Left, TUNEL staining of organoids after 60-day culture with and without Aβ treatment. Right, quantification of $TUNEL^{+}/DAPI^{+}$ cells in hCOs and mhCOs at days 60 with and without Aβ treatment. Data represent the mean ± SEM (n=7 organoids from three independent differentiation replicates of two hESCs lines). The dash lines indicate the surface of the samples. The scale bar represents 100 μm.

Supplementary Fig 9

a

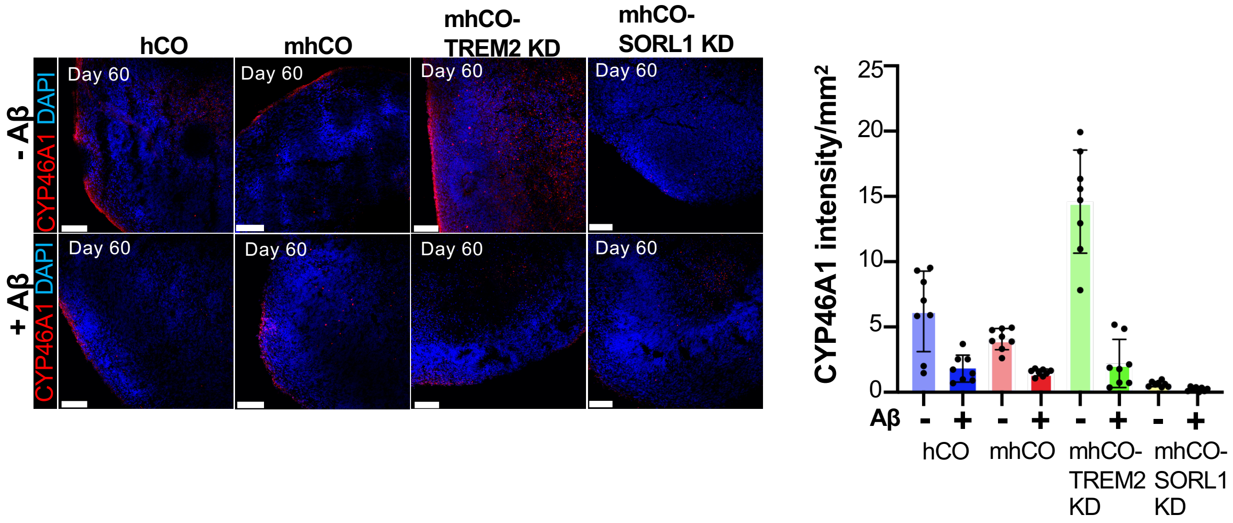

b

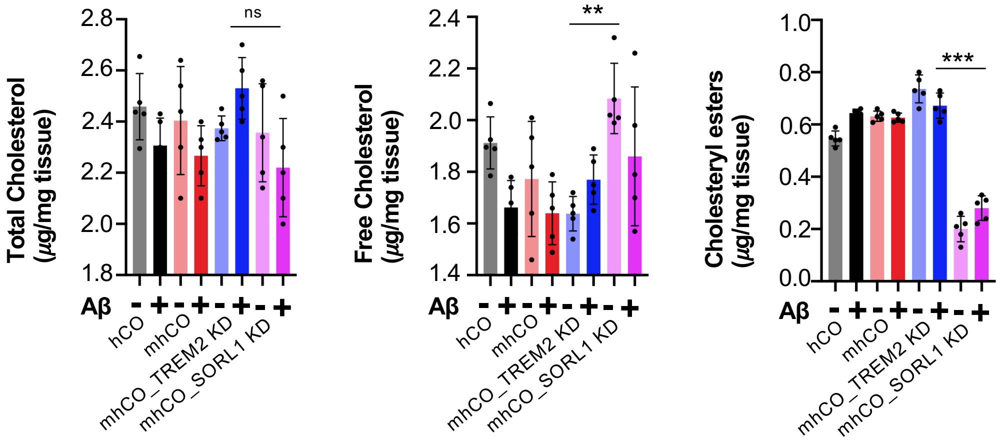

Supplementary Fig. 9. Cholesterol turnover in mhCO variants. a, Left, CYP46A1 staining of organoids after 60-day culture with and without Aβ treatment. Right, quantification of CYP46A1$^+$ signal intensity per mm^2 in hCOs and mhCOs at days 60 with and without Aβ treatment. Data represent the mean ± SEM (n=8 organoids from three independent differentiation replicates of a hESCs line). The scale bar represents 50 μm. **b,** Total cholesterol (left), free cholesterol (middle), and cholesteryl esters concentrations (right, μg/mg tissue) in the whole brain organoid variants with and without Aβ treatment. Data represent the mean ± SEM (n=5 organoids from three independent differentiation replicates of two hESCs lines, **p=0.004822, ***p=0.000151). The unpaired two-tailed t-test was used for all comparisons.

Supplementary Table 1. gRNA library used for the CROP-seq AD-linked genes screen.

Gene	Target Sequence
TREM2-sg1	GAAAGACGAGATCTTGCACA
TREM2-sg2	CGCCTTCATAATTCACCCCA
CD33-sg1	GGGGAGTTCTTGTCGTAGTA
CD33-sg2	GACAAGAACTCCCCAGTTCA
SORL1-sg1	CAGTAGCGTTCGCCCGAACA
SORL1-sg2	CGCTGCACATTCTCTCCTGG
APOE4-sg1	AGGACGTCCTTCACCTCCGC
APOE4-sg2	AGGGTCCCAGCTCTTTCTAG
PICALM-sg1	TTAGAATGGCAGCAACGTGT
SHIP1-sg1	GAGCCGGTCATTCCACCCAG
CD2AP-sg1	AGTGCTAAGGAAGAGGCGAG
RIN3-sg1	ATCATGCCGCCGGCAGCTCC
BIN1-sg1	AAGGCAGCTTATTGTCCGGA
PLCG2-sg1	GAAGCAGAAGTAGCGAGCGC
CASS4-sg1	CAGGCATTGAGACGTGAGTG
PTK2B-sg1	AGGTAGGTGTGCAACGGCTC

Supplementary Table 2. List of antibodies used for Immunostaining.

Antibody	Source	Identifier
IBA1	Wako	Cat# 019-19741
CD68	Abcam	Cat# ab31630
CD68	Abcam	Cat# ab199000
C1qC	R&D Systems	Cat# AF3337
TMEM119	Atlas Antibodies	Cat# AMAb91528
PU.1	ThermoFisher	Cat# Ma5-15064
CSF1R	ThermoFisher	Cat# Pa5-25974
LAMP1	Cell Signaling	Cat# 15665S
P2RY12 (4H5L19)	ThermoFisher	Cat# 702516
β-Amyloid (D54D2)	Cell Signaling	Cat# 8243S
Ki67	BD Biosciences	Cat# 556003
KRT14	Abcam	Cat# ab9220
Tp63	Fitzgerald	Cat# 70R-50620
GFP	Sigma	Cat# SAB4600051
MAP2	Millipore	Cat# MAB3418
PSD95	Abcam	Cat# ab12093
TBR2	Abcam	Cat# ab23345
SATB2	Abcam	Cat# ab51502
CTIP2 (25B6)	Abcam	Cat# ab18465
SOX2	Cell Signaling	Cat# 3579

Supplementary Table 3. List of primers used for qPCR experiment.

Gene	Forward Primer	Reverse primer
PU.1	CGACCATTACTGGGACTTCCA	GGAGCTCCGTGAAGTTGTTCTC
IBA1	GTCCCTGAAACGAATGCTG	CCTTTTCTCTCGCTTTTTCCTC
LAG3	CGTCTCCATCATGTATAACCTC	GTAAAGTCGCCATTGTCTCC
CX3CR1	CAACAGCAAGAAGCCCAAG	CGATGAAGAAGAAGGCGGTAG
CD11b	TCCAGAACAACCCTAACCC	CGCCAAACTTTTCTCCATCC
TMEM119	GCCTCCTCATCCTTCTGTTG	AAGAAGTCCACTATCCCATCC
P2RY12	ACATCCAACCCCAAAAATCTC	TTTACCTACACCCCTCGTTC
SALL1	TCACAACCTCTCTACCTCAAC	TGCATTCTGAGAAGCCAAC
GPR34	CCAACCGCCACAAAACTTC	TATGTTCCCAACCAGTCCC
AXL	CCATCCTCACACCCCTTATC	TCTTGCCTTAGCCCTATGTC
CSF1R	CACCAAGCTCGCAATCCCTC	CTCTACCACCCGGAAGAACA
MPO	ACCATCCGCAACCAGATCAA	GCTCCTCGCTGCCGTACA
IL3RA	CCGCATCCCTCACATGAAA	TCCCAGACCACCAGCTTGTC
CD33	CACTTTCTTCCATCCCATACC	TGAACCATTATCCCTCCTCC
SORL1	AAACTGCCCTACCACCATC	ACTTTCATCACTGTTGTCTCC
TREM2	CACCCACTTCCATCCTTCTCC	TCCAGTTCACTGGGTGGATG
TP63	TATAACACAGACCACGCGCAGA	GTGATGGAGAGAGAGCATCGAAG
SOX9	GAGGAAGTCGGTGAAGAACG	GTTTTGGGGGTGGTGGGT
CD45	GACACGGCTGACTTCCAGAT	CACTGGGCATCTTTGCTGTA
POU5F1 (OCT4)	CCTCACTTCACTGCACTGTA	CAGGTTTTCTTTCCCTAGCT
TUJ1	GCCGCTACCTGACGGTGGC	GGGCGGGATGTCACACACGG
