# Supplementary material for: Expression of the transcription factor PU.1 induces the generation of microglia-like cells in human cortical organoids
Source: Nat Commun. 2022 Jan 20;13:430. doi: 10.1038/s41467-022-28043-y (PMC8776770; doi:10.1038/s41467-022-28043-y)
Supplement: Supplementary file 2 — Description of Additional Supplementary Files [file 41467_2022_28043_MOESM2_ESM.docx]

Description of Additional Supplementary Files

Title: Supplementary Movie 1

Description: Whole-mount immunostaining of control hCOs at day 90 for IBA1, PSD95, and MAP2. Confocal z-stack movie of a whole cortical organoid, containing MAP2+ neurons and ramified microglia-like cells. DAPI (Blue), IBA1 (Red) MAP2 (Green) and PSD95 (Magenta). (n=5, from two independent batches).

Title: Supplementary Movie 2

Description: Whole-mount immunostaining of mhCOs at day 90 for IBA1, PSD95, and MAP2. Confocal z-stack movie of a whole cortical organoid, containing MAP2+ neurons and ramified microglia-like cells. DAPI (Blue), IBA1 (Red) MAP2 (Green) and PSD95 (Magenta). (n=5, from two independent batches).

Title: Supplementary Movie 3

Description: Time lapse series of imaging showing the typical motility of microglia-like cells in the mhCOs. Time is shown in hour:min:s.

Title: Supplementary Movie 4

Description: Time lapse series of imaging showing the GFP microglia-like cells engulfing Aβ-oligos (red) in the mhCOs. Time is shown in hour:min:s.

Title: Supplementary Movie 5

Description: Co-Immunostaining for CD68 and Aβ in Aβ-treated control hCOs at day 90. Confocal z-stack movie of a sectioned cortical organoid, showing Aβ-treated organoids stained for phagocytic marker CD68 and Aβ. DAPI (Blue), Aβ (Red), and CD68 (Green).

Title: Supplementary Movie 6

Description: Co-Immunostaining for CD68 and Aβ in Aβ-treated mhCOs at day 90. Confocal z-stack movie of a sectioned cortical organoid, showing Aβ-treated organoids stained for phagocytic marker CD68 and Aβ. DAPI (Blue), Aβ (Red), and CD68 (Green).
