# Supplementary material for: Expression of the transcription factor PU.1 induces the generation of microglia-like cells in human cortical organoids
Source: Nat Commun. 2022 Jan 20;13:430. doi: 10.1038/s41467-022-28043-y (PMC8776770; doi:10.1038/s41467-022-28043-y)
Supplement: Supplementary file 9 — Reporting summary [file 41467_2022_28043_MOESM9_ESM.pdf]

nature research

Corresponding author(s): In-Hyun Park
Last updated by author(s): Dec 8, 2021

Reporting Summary

Nature Research wishes to improve the reproducibility of the work that we publish. This form provides structure for consistency and transparency in reporting. For further information on Nature Research policies, see our Editorial Policies and the Editorial Policy Checklist.

Statistics

For all statistical analyses, confirm that the following items are present in the figure legend, table legend, main text, or Methods section.

n/a	Confirmed	
☐	☒	The exact sample size (*n*) for each experimental group/condition, given as a discrete number and unit of measurement
☐	☒	A statement on whether measurements were taken from distinct samples or whether the same sample was measured repeatedly
☐	☒	The statistical test(s) used AND whether they are one- or two-sided<br>*Only common tests should be described solely by name; describe more complex techniques in the Methods section.*
☒	☐	A description of all covariates tested
☒	☐	A description of any assumptions or corrections, such as tests of normality and adjustment for multiple comparisons
☐	☒	A full description of the statistical parameters including central tendency (e.g. means) or other basic estimates (e.g. regression coefficient) AND variation (e.g. standard deviation) or associated estimates of uncertainty (e.g. confidence intervals)
☐	☒	For null hypothesis testing, the test statistic (e.g. *F*, *t*, *r*) with confidence intervals, effect sizes, degrees of freedom and *P* value noted<br>*Give P values as exact values whenever suitable.*
☒	☐	For Bayesian analysis, information on the choice of priors and Markov chain Monte Carlo settings
☒	☐	For hierarchical and complex designs, identification of the appropriate level for tests and full reporting of outcomes
☐	☒	Estimates of effect sizes (e.g. Cohen's *d*, Pearson's *r*), indicating how they were calculated

Our web collection on statistics for biologists contains articles on many of the points above.

Software and code

Policy information about availability of computer code

Data collection	Immunostaining images were captured using LAS AF (Leica) 4.0 or Qcapture Pro 7 (QICAM).
Data analysis	Software used to analyze data was GraphPad Prism Version 8.2.0 for graphs and statistics. ImageJ 1.51h was used for quantification of signals in microglia-like cells and organoids. Cellranger (v3.0.2) was used for alignment and initial QC of scRNA-seq. Seurat (v3.0.2) was used for normalization and visualization of scRNA-seq. GOstats (v2.46.0) was used for Gene Ontology analysis. GSEAPY (v0.9.3) was used for enrichment analysis of cell type-specific gene signatures. Monocle (v2.99.3) was used for differentiation trajectory analysis. GSEA (v2.2.2) was used to evaluate disease state of neurons. CROP-seq reads were first aligned to gRNA plasmid library sequences by Bowtie2 (v2.3.0)

For manuscripts utilizing custom algorithms or software that are central to the research but not yet described in published literature, software must be made available to editors and reviewers. We strongly encourage code deposition in a community repository (e.g. GitHub). See the Nature Research guidelines for submitting code & software for further information.

Data

Policy information about availability of data

All manuscripts must include a data availability statement. This statement should provide the following information, where applicable:
- Accession codes, unique identifiers, or web links for publicly available datasets
- A list of figures that have associated raw data
- A description of any restrictions on data availability

All relevant data supporting this study are available from the corresponding authors upon reasonable request. Single-cell transcriptome data are available in the Gene Expression Omnibus under accession code GSE175722.

Field-specific reporting

Please select the one below that is the best fit for your research. If you are not sure, read the appropriate sections before making your selection.

[x] Life sciences [ ] Behavioural & social sciences [ ] Ecological, evolutionary & environmental sciences

For a reference copy of the document with all sections, see nature.com/documents/nr-reporting-summary-flat.pdf

Life sciences study design

All studies must disclose on these points even when the disclosure is negative.

Sample size	Samples sizes were determined based on the means and variations observed in our pilot experiments.
Data exclusions	No data were excluded.
Replication	The key findings in the study were reliably reproduced in several independent cohorts. Most experiments were performed using 2 human embryonic stem cell lines. For each analysis, multiple organoids from more than one differentiation were used. Replicates for each type of experiment are indicated in the figure legends.
Randomization	hCOs and mhCOs of similar diameter were randomly selected for each type of experiment. At least 3-5 organoids from 2 different hESCs differentiated in independent batches were used in each experiment.
Blinding	The study involved unbiased quantification and analysis for immunostaining, 3D imaging, gene expression data sets, and electrophysiology.

Reporting for specific materials, systems and methods

We require information from authors about some types of materials, experimental systems and methods used in many studies. Here, indicate whether each material, system or method listed is relevant to your study. If you are not sure if a list item applies to your research, read the appropriate section before selecting a response.

Materials & experimental systems

n/a	Involved in the study
[ ]	[x] Antibodies
[ ]	[x] Eukaryotic cell lines
[x]	[ ] Palaeontology and archaeology
[ ]	[x] Animals and other organisms
[ ]	[x] Human research participants
[x]	[ ] Clinical data
[x]	[ ] Dual use research of concern

Methods

n/a	Involved in the study
[x]	[ ] ChIP-seq
[x]	[ ] Flow cytometry
[x]	[ ] MRI-based neuroimaging

Antibodies

Antibodies used

Immunostaining:
Mouse anti-MAP2 (Millipore, MAB3418, Lot.3129246). Used at 1:100.
Mouse anti-Sox2 (Millipore, MAB4423, Lot.2707795). Used at 1:100.
Mouse anti-SATB2(Abcam, Ab51502). Used at 1:200.
Rat anti-CTIP2 (Abcam, Ab18465, Lot.GR322373). Used at 1:200.
Mouse anti-Amyloid-beta(D54D2) (Cell Signaling, 8243S, Lot.2707795). Used at 1:200.
Mouse anti-CD68 (Abcam, ab31630, Lot.GR3277010-4). Used at 1:50.
Rabbit anti-TBR2 (Abcam, ab23345, Lot.GR306193-2). Used at 1:50.
Goat anti-PSD95 (Abcam, ab12093, Lot.GR317630-5). Used at 1:50.
Rabbit anti-IBA1 (Wako, 019-19741, Lot.PTG5394). Used at 1:50.
Mouse anti-CD68 (Abcam, ab199000, Lot.GR3296263-1). Used at 1:50.
Rabbit anti-P2RY12 (Invitrogen, (4H5L19) Cat.702516, Lot.2296315). Used at 1:200.
Rabbit anti-PU.1 (Invitrogen, (E.388.3) Cat.MA5-15064, Lot.VH3048379A). Used at 1:100.
Mouse anti-TMEM119 (Atlas antibodies, AMAb91528, Lot. MAB-03452, Clone ID. CL8714). Used at 1:100.
Mouse anti-LAMP1 (Cell Signaling, Cat.15665, Lot.1).Used at 1:100.
Rabbit anti-CSFI1R (Invitrogen Cat.PA5-25974, Lot.VK2962977). Used at 1:200.
Rabbit anti-CYP46 (NOVUS, NB400-140, Lot.A2). Used at 1:200.
Rabbit anti-AXL (C89E7) (Cell Signaling, Cat.8661S, Lot. 7). Used at 1:400.
Rabbit anti-Cas3 (Cell Signaling, Cat.9661s, Lot 47). Used at 1:100.
Rabbit anti-NRXN1 (Novus Biologicals, Cat. NBP2-68920, Lot. R98003). Used at 1:50.

	Mouse anti-MAP2 (Millipore, Cat. MAB3418, Lot, 3607062, Clone AP20). Used at 1:100.<br>Mouse anti-Ki67 (BD biosciences, Cat. 556003, Lot,8239549 , Clone B56). Used at 1:100.<br>Data also provided in the manuscript : In supplementary table 2, we have listed all the antibodies used in this study and included the resource (i.e. company, catalog no..etc).
Validation	Anti-SATB2 antibody [SATBA4B10] - C-terminal (ab51502) validated in human neural 2D and 3D cultures in previous publications (Nat Methods 16:75-78 (2019), Nature 556:370-375 (2018)).<br>Anti-MAP2 Antibody (clone AP20, Millipore) validated in human neural cells in multiple publications (Nature communications 6 6340 2015, Nature 507 99-103 2014).<br>Anti-CTIP2 (Abcam, Ab18465) validated in human neural 2D and 3D cultures in previous publications (Neuron 101:459-471.e5 (2019), Nature 563:691-695 (2018)).<br>Anti-Amyloid-beta(D54D2) (Cell Signaling, 8243S validated in human neural cells in multiple publications (Neuron on 27 June 2018 by Lin, Y. T., Seo, J., et al.., Nature Communications on 6 May 2016 by Beck, S. J., Guo, L., et al..).<br>Anti-CD68 (Abcam, ab31630) validated in human microglia cells in multiple publications (Nat Commun 10:1463 (2019), Nat Commun 10:3473 (2019), Nat Commun 9:551 (2018), Proc Natl Acad Sci U S A 114:E75-E84 (2017)).<br>Anti-TBR2 (Abcam, ab23345) validated in human neural cells in multiple publications (Cell 174:590-606.e21 (2018), Nature 551:227-231 (2017)).<br>Anti-PSD95 (Abcam, ab12093) validated in human neural cells in multiple publications (Brain 135:2155-68 (2012), Nat Protoc 9:1682-97 (2014)).<br>Anti-IBA1 (Wako, 019-19741) validated in human immune cells in multiple publications (Neuron. 2018 Oct 10;100(1):120-134.e6, Nature. 2018 Oct;562(7728):578-582).<br>Anti-CD68 (Abcam, ab199000) validated in human brain microglia cells in a publication (Clin Cancer Res. 2019 Jun 15;25 (12):3643-3657).<br>Anti-Pu.1 (Invitrogen, (E.388.3) Cat.MA5-15064) validated in human T-cells in a publication (Nature Immunol. 2014 Jul 15(7):676-86).<br>Anti-LAMP1 (Cell Signaling, Cat.15665) validated in human myeloid cells in multiple publications (Elife, 2020 Nov 2;9:e55547, Nat Commun. 2019 Sep 20;10(1):4320).<br>Anti-CYP46 (NOVUS, NB400-140) validated in human neural cells in multiple publications (J Biol Chem. 2004 Aug 13;279 (33):34674-81, Toxicology 2020 Feb 28;432:152381).<br>Anti-AXL (Cell Signaling, Cat.8661S) validated in human cells in multiple publications (Mol Cell. 2020 Jan 2;77(1):120-137.e9,Cell Rep.2019 Dec 10;29(11):3421-3434.e8,Nat Commun.2019 Apr 3;10(1):1515).<br>Anti-Cas3 (Cell Signaling, Cat.8661S) validated in human neural 2D and 3D cultures in previous publications (Elife. 2021 Jan 22;10:e62387., Cell Rep. 2020 Dec 8;33(10):108447,Nat Commun. 2020 Nov 2;11(1):5540).<br>Anti-NRXN1 (Novus Biologicals, Cat. NBP2-68920) used in this study.<br>Anti-MAP2 (Millipore, Cat. MAB3418) validated in human neural cells in multiple publications (Nat Commun. 2015 Mar 20;6:6340,Nat Med. 2014 Oct;20(10):1157-6).<br>Anti-Ki67 (BD biosciences, Cat. 556003) validated in human cells in multiple publications (J Exp Med. 2011; 208(2):227-234, J Exp Med. 2009; 206(9):2013-2025)<br>Anti-NRXN1 (Novus Biologicals, Cat. NBP2-68920) has not reported in any publication yet.

Eukaryotic cell lines

Policy information about cell lines

Cell line source(s)	Our collaborated generated HES-3 NKX2-1GFP/w hESCs and provided us and we reported in Xiang et al., 2017, Cell Stem Cell.<br>HES-3 NKX2-1GFP/w hESCs<br>HES-3-TRE3G-dCAS9-mCherry/AAVS1-CAG-rTTA- hESCs (named BC4 lines)<br>HES-3-TRE3G-PU.1-IRES-eGFP-rTTA- hESCs (named BC61 lines)
Authentication	Immunostaining for pluripotent markers, teratoma formation in mice, on-target and off-target sequence analysis were performed to authenticate.
Mycoplasma contamination	All cell lines were confirmed as mycoplasma negative before experiments.
Commonly misidentified lines<br>(See ICLAC register)	No commonly misidentified line was used.

Animals and other organisms

Policy information about studies involving animals; ARRIVE guidelines recommended for reporting animal research

Laboratory animals	The Rag2-/- GammaC-/- mice were obtained from the Jackson Laboratory. Both male and female (2 each) animals at postnatal day 4 were used for injections.
Wild animals	The study did not involve wild animals.
Field-collected samples	The study did not involve field-collected samples
Ethics oversight	All animal experiments described in this study were approved by the Institutional Animal Care & Use Committee of Yale University and were performed under the guidelines of Yale University Institutional Animal Care and Use Committee with approved protocol (no. 2016–11347).

Note that full information on the approval of the study protocol must also be provided in the manuscript.

Human research participants

Policy information about studies involving human research participants

Population characteristics	*Describe the covariate-relevant population characteristics of the human research participants (e.g. age, gender, genotypic information, past and current diagnosis and treatment categories). If you filled out the behavioural & social sciences study design questions and have nothing to add here, write "See above."*
Recruitment	*Describe how participants were recruited. Outline any potential self-selection bias or other biases that may be present and how these are likely to impact results.*
Ethics oversight	*Identify the organization(s) that approved the study protocol.*

Note that full information on the approval of the study protocol must also be provided in the manuscript.
